# NanoVar: accurate characterization of patients’ genomic structural variants using low-depth nanopore sequencing

**DOI:** 10.1186/s13059-020-01968-7

**Published:** 2020-03-03

**Authors:** Cheng Yong Tham, Roberto Tirado-Magallanes, Yufen Goh, Melissa J. Fullwood, Bryan T.H. Koh, Wilson Wang, Chin Hin Ng, Wee Joo Chng, Alexandre Thiery, Daniel G. Tenen, Touati Benoukraf

**Affiliations:** 1 Cancer Science Institute of Singapore, National University of Singapore, Centre for Translational Medicine, 14 Medical Drive, #12-01, Singapore, 117599 Singapore; 2 School of Biological Sciences, Nanyang Technological University, Singapore, 637551 Singapore; 3 Department of Orthopedic Surgery, National University Health Systems, Singapore, 119228 Singapore; 4 Department of Orthopaedic Surgery, Yong Loo Lin School of Medicine, National University of Singapore, Singapore, 119228 Singapore; 5 Department of Hematology-Oncology, National University Cancer Institute of Singapore, National University Health System, Singapore, 119228 Singapore; 6 Department of Medicine, Yong Loo Lin School of Medicine, National University of Singapore, Singapore, 119228 Singapore; 7 Department of Statistics and Applied Probability, National University of Singapore, Singapore, 117546 Singapore; 8 Harvard Stem Cell Institute, Harvard Medical School, Boston, MA 02115 USA; 9 Discipline of Genetics, Faculty of Medicine, Memorial University of Newfoundland, St. John’s, NL A1B 3V6 Canada

**Keywords:** Structural variants, SV characterization, Long reads, Oxford Nanopore sequencing, Third-generation sequencing, WGS, Low depth

## Abstract

The recent advent of third-generation sequencing technologies brings promise for better characterization of genomic structural variants by virtue of having longer reads. However, long-read applications are still constrained by their high sequencing error rates and low sequencing throughput. Here, we present NanoVar, an optimized structural variant caller utilizing low-depth (8X) whole-genome sequencing data generated by Oxford Nanopore Technologies. NanoVar exhibits higher structural variant calling accuracy when benchmarked against current tools using low-depth simulated datasets. In patient samples, we successfully validate structural variants characterized by NanoVar and uncover normal alternative sequences or alleles which are present in healthy individuals.

## Background

Structural variations are implicated in the development of many human diseases [1, 2] and account for most of the genetic variations by means of nucleotides in the human population [3, 4]. Structural variants (SVs), defined as genomic alterations greater than 50 base pairs (bp) [5], can functionally affect cellular physiology by forming genetic lesions which may lead to gene dysregulation or novel gene fusions, driving the development of diseases such as cancer [6, 7], Mendelian disorders [8, 9], and complex diseases [10]. SVs can exist as different classes including deletion, duplication, insertion, inversion, and translocation. Over the years, disease-associated SVs were indicated as biomarkers for diagnosis [9, 11], prognosis [12], and therapy guidance for patients [13], which could be screened through sequencing-based and non-sequencing-based methods in clinics. As the clinical impacts of SVs continue to unveil, there is a clear need for accurate, rapid, and inexpensive workflows for routine SV profiling in patients to expedite biomarker discovery and broaden clinical investigations [7].

There are currently two main standards of sequencing-based methods for comprehensive SV detection: long-read or third-generation sequencing (3GS) and short-read or second-generation sequencing (2GS). Although 3GS technologies were made accessible to a large audience, it has not yet supplanted 2GS technologies due to its higher sequencing error rate and lower throughput [14]. While 3GS is currently mainly restricted to the study of small genomes [15] or targeted sequencing [16], recent studies have reported mammalian whole-genome sequencing (WGS) [17, 18] but at a higher sequencing cost per megabase as compared to the older technologies. In the domain of SV discovery, many groups have reported that 3GS approaches provided higher SV detection sensitivity and resolution than 2GS, despite their higher sequencing error rate [9, 11, 19]. This is mostly due to the inadequacy of short reads (50–200 bp) to elucidate large genomic variations also involving novel sequence insertions or repetitive elements, which may give rise to high false discovery rates [5, 20]. On the other hand, longer read lengths (> 1 kb) reduce mapping ambiguity, resolve repetitive sequences [21] and complex SVs [8], and discover a much larger extent of SVs than short reads [17, 18]. Despite better SV detection capabilities, the low throughput and high sequencing cost per megabase of 3GS obstruct its feasibility to be used in routine SV interrogation in patients.

To overcome these issues, we developed a new SV caller tool, NanoVar, which utilizes low-depth Oxford Nanopore Technologies (ONT) WGS data for accurate SV characterization in patients. NanoVar adopts a neural-network-based algorithm for high-confident SV detection and SV zygosity estimation for all SV classes. It is optimized to work with shallow long-read WGS data at a minimum sequencing depth of 4X (12 gigabases (Gb)) for homozygous SVs and 8X (24 Gb) for heterozygous SVs, which can be achieved with one to ten ONT MinION sequencing runs, depending on the flowcell chemistry, library preparation kit, and sample quality. In this manuscript, we evaluated NanoVar’s SV detection precision and recall among other tools using simulation datasets and real data. When applied to patient data, we demonstrated the feasibility and speed of implementing the NanoVar workflow for SV discovery in low-depth 3GS clinical samples.

## Results

### The NanoVar workflow

**Fig. 1:**
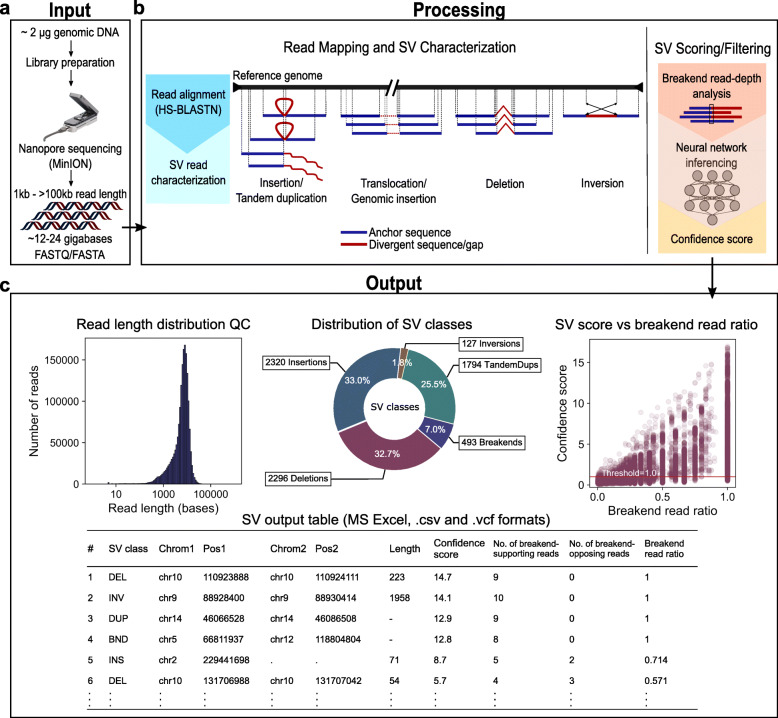
The NanoVar workflow. **a** About 2 μg of human genomic DNA is set aside for library preparation and nanopore sequencing to generate 3GS long sequencing reads. Long reads from all sequencing runs are combined into a single FASTQ/FASTA file (at least 12 Gb, recommended 24 Gb) which is used as input into NanoVar. **b** NanoVar SV characterization process. (Left) Long reads are aligned to a reference genome using HS-BLASTN to identify anchor sequences (blue) and divergent sequences or gaps (red) within each read. Next, the alignment information is used to detect and characterize the different SV classes. (Right) For each characterized SV, read-depth coverage is calculated at their breakend(s) site for the number of breakend-supporting and breakend-opposing reads. The breakend read depth, together with other alignment information, is employed as features in a neural network model to infer a confidence score for each SV. **c** NanoVar outputs all characterized SVs in a VCF file and produces an HTML report for QC and result visualization. The following figures can be found in the HTML report. (Top-left) Histogram showing the length distribution QC of the input sequencing reads. (Top-middle) Donut chart showing the distribution of SV classes characterized in the dataset (after confidence score filtering). Breakends represent translocation or genomic insertion SV. (Top-right) Scatter plot displaying the confidence score and breakend read ratio (fraction of breakend-supporting reads at a breakend) of each SV, also showing the confidence score threshold parameter used for filtering (red line). (Bottom) Table showing the details of all characterized SVs, which can be sorted, filtered, and extracted in CSV or MS Excel formats

The NanoVar workflow is a series of processes that utilizes 3GS long reads to discover and characterize SVs in DNA samples. The sequencing of the genome of interest is carried out by ONT MinION to produce long reads reported in FASTQ/FASTA formats (Fig. 1a). The sequencing output of several sequencing runs can be combined to achieve enough depth of coverage for SV discovery. Based on our initial tests performed in simulated datasets (cf. hereafter), we recommend having at least 12–24 Gb of sequencing data (covering approximately 4–8X of the human genome), which can be achieved through one to ten MinION runs depending on the flowcell chemistry (R9.4, R9.5), library preparation kit (1D, 1D^2^, 2D), and DNA sample quality (purity, quantity, and fragment length). The R9.4 flowcell chemistry with a 1D sequencing library using high purity DNA with a mean fragment length of 8 kb provides the highest throughput per MinION flowcell (above 12 Gb). The combined FASTQ/FASTA file is used as input into the NanoVar tool for SV processing. NanoVar begins by mapping the long reads against a reference genome using HS-BLASTN [22] to obtain the alignment profile of each read (Fig. 1b). Reads with incomplete alignments (containing divergent sequence/gap) are selected and evaluated through an SV characterization algorithm (Additional file 1: Fig. S1) to characterize for the possible SV classes. NanoVar can distinguish six classes of SV: deletion (DEL), inversion (INV), tandem duplication (DUP), insertion (INS, novel sequence insertion/insertion of sequences absent from reference genome), transposition (insertion of sequences found elsewhere in the reference genome), and translocation. Due to the close resemblance in altered sequences between a transposition and a translocation, they are collectively labeled as “breakends” (BND) but are still dissociable by the “SV2” field in the “INFO” column of the variant calling format (VCF) file. After all reads are classified, NanoVar calculates the read-depth coverages for all the SV breakend sites, separating the number of breakend-supporting reads and breakend-opposing reads. Lastly, the read-depth coverage of each SV, together with other SV characteristics, are used as features for a simulation-trained neural network classifier to determine a confidence score for each SV. This confidence score is used to rank the SVs by confidence and reduces false positives in the final output. The filtered list of SVs is recorded in a VCF file and an HTML report. The HTML report provides an overview of the SV analysis and an SV output table containing the information of each SV which can be filtered and downloaded in MS Excel or CSV formats. The figures presented in the report also include an SV class distribution chart and read length distribution of the sequencing reads which serves to QC for the input (Fig. 1c). NanoVar also assigns a breakend read ratio value to each SV to estimate their SV zygosity, where a ratio of 1.0 refers to a homozygous estimation and 0.5 refers to a heterozygous estimation.

### Benchmarking NanoVar using simulations

**Fig. 2:**
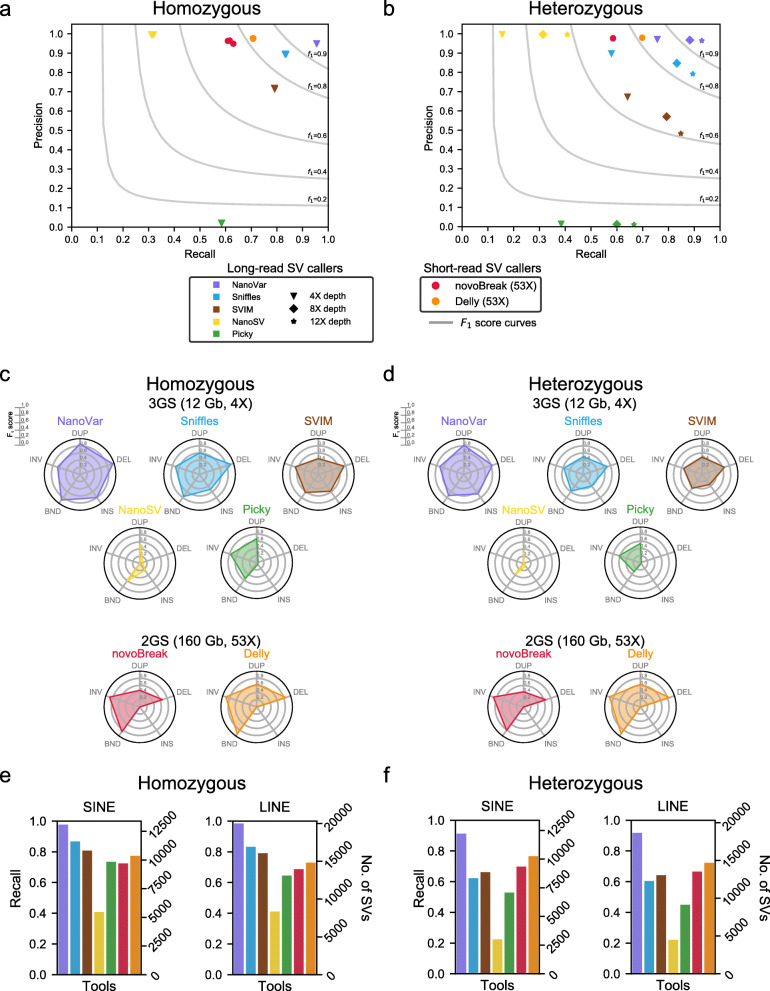
NanoVar performance benchmarking. **a**, **b** SV breakend precision and recall by SV caller tools in simulation data with homozygous or heterozygous SVs. There are three homozygous SV datasets with 42,000 SVs each and one heterozygous SV dataset with 42,000 SVs at different sequencing depths. For tools with SV scoring, the optimal score was selected for them based on the *F*_1_ score at 4X sequencing depth (NanoVar, 1.0; NanoSV, 0; SVIM, 0; novoBreak, 27.5). The markers of different tools are separated by color, while different sequencing depths are separated by shapes. **b**, **c** Radar charts showing the *F*_1_ scores for each SV class characterized by each tool for homozygous and heterozygous SVs in simulation 1. DUP tandem duplication, DEL deletion, INS insertion, BND breakend, INV inversion. We presented the tools separately according to their utilization of 3GS and 2GS data. SV class annotation evaluation was included in this analysis. **e**, **f** Recall of homozygous and heterozygous SVs intersecting with SINE and LINE regions as detected by the different tools. The tools are separated by the same color code as the other plots in this figure. SV analysis of the other repetitive sequence families can be found in Additional file 1: Fig. S3

To evaluate NanoVar’s performance among other existing SV callers, we utilized a homozygous SV simulated dataset (triplicate) and a heterozygous SV simulated dataset (doi: 10.5281/zenodo.3569479). Each dataset contains 42,000 SVs of the various SV classes with sizes estimated from real data (Additional file 1: Fig. S2a). Most of the SV breakends (> 75%) are positioned within repetitive sequence regions demarcated by UCSC Browser’s RepeatMasker track. Sequencing reads of both long-read and short-read sequencing were simulated to a sequencing depth of 4–12X and 53X respectively and were used as input for the workflows of each tool. Sniffles [19], SVIM [23], Picky [24], and NanoSV [25] are 3GS long-read SV callers while novoBreak [26] and Delly [27] are 2GS short-read SV callers. To evaluate predicted SVs fairly, we segregated each ground-truth SV into its breakend coordinates and allow a 400-bp error distance around it to create an 800-bp window. Each DEL, INV, and DUP SV possesses two breakends, while each INS and BND SV possesses one breakend. SVs predicted by each tool were also segregated to SV breakends and intersected with the ground-truth breakend regions. A hit will be considered if a predicted SV breakend falls within any ground-truth breakend region. We first performed a global SV breakend evaluation where SV class prediction was omitted (Fig. 2a, b, Additional file 1: Table S1). At a sequencing depth of 4X, NanoVar outperformed the other 3GS tools in precision and recall for homozygous and heterozygous SVs, achieving the highest *F*_1_ scores of 0.95 and 0.85 respectively. Sniffles and SVIM followed close behind with *F*_1_ scores of 0.86 and 0.75 for homozygous SVs and 0.70 and 0.66 for heterozygous SVs. For both SV zygosities, NanoSV achieved the highest precision of more than 0.99 among all tools, but it displayed low detection sensitivities (0.31, 0.16) which brought its *F*_1_ scores down to 0.48 and 0.27. Conversely, Picky performed slightly better in recall (0.58, 0.38) but exhibited low precision (0.02, 0.02). While NanoVar, Sniffles, and SVIM coped reasonably well with 4X sequencing data, such sequencing depth might not be appropriate for NanoSV and Picky. To find out if a higher sequencing depth might improve performance, we tested the 3GS tools at sequencing depths of 8X and 12X for heterozygous SVs and observed a comparable increase in recall for all tools (Fig. 2b). Despite the increase in recall, *F*_1_ score improvement was only seen in NanoVar and NanoSV as the other tools had a decrease in precision as coverage increases. The performance of NanoSV might continue to improve with an increasing depth of coverage as it was previously observed to perform better with high coverage (79X) ONT real dataset [28]. Similar to 4X depth, NanoVar exhibited the highest *F*_1_ scores of 0.92 and 0.95 at 8X and 12X depths respectively. In general, when compared to 2GS SV callers such as Delly and novoBreak, 3GS SV callers recalled more SVs for both zygosity states, despite using considerably lower depth of sequencing data.

Next, we carried out separate breakend evaluation for the different SV classes to investigate the SV class characterization accuracy of each tool at 4X depth for 3GS and 53X depth for 2GS (Fig. 2c, d; Additional file 1: Table S2). A hit is only considered when a predicted SV breakend falls into a ground-truth breakend region that belongs to the same SV class as the predicted. NanoVar portrayed relatively well-rounded accuracies for all the SV classes for homozygous SVs (DUP 0.85, DEL 0.96, INS 0.80, BND 0.88, INV 0.66) and heterozygous SVs (DUP 0.81, DEL 0.83, INS 0.67, BND 0.73, INV 0.70), exhibiting the best accuracies for DUP and INS among all tools. Sniffles and SVIM also performed moderately well, with their highest *F*_1_ scores pertaining to homozygous DEL (0.91, 0.75) and lowest *F*_1_ scores pertaining to heterozygous INS (0.40, 0.35). The low DEL and INS accuracies observed in Picky were attributed to its high false-positive rates when calling for these two classes (precision < 0.03), suggesting an explanation for its poor overall performance in the first evaluation. Despite that, Picky performed considerably well for DUP, INV, and BND SVs. NanoSV’s limited performance was due to its inability to characterize INV SVs completely and its low SV recall which was prominently observed in DEL and INS, despite its capability of being highly precise. Both 2GS SV callers performed acceptably well especially for INV SVs, which excelled over all other tools. However, they were inadequate in characterizing INS SVs when compared to most 3GS SV callers.

As SV formation is known to be associated with repeat regions in the human genome, we investigated the effects of repetitive sequences on SV recall by all tools within our simulations (Fig. 2e, f, Additional file 1: Fig. S3). We intersected all ground-truth SV regions with the RepeatMasker track from UCSC’s Browser to identify their overlaps with the different repetitive sequence families such as short interspersed nuclear elements (SINE) and long interspersed nuclear elements (LINE). In our dataset, most of the ground-truth SVs overlapped with SINE and LINE regions, while less were scattered among DNA transposons, long terminal repeats (LTR), low complexity, satellite, and simple repeat regions. For all tools, SV recall was observed to be fairly consistent across the different repeat families, suggesting that neither of the tools was greatly vulnerable to any specific type of repetitive sequence. As expected with regard to the previous SV breakend evaluations, NanoVar displayed the greatest recall of SVs across all kinds of repeats for both zygosity states. Taken together, our simulation results demonstrated NanoVar’s higher SV characterization sensitivity and precision among other long-read 3GS SV callers at a sequencing depth of 4–12X.

### Benchmarking NanoVar using real data

As simulated data may not capture all aspects of reality, we also carried out benchmarking using real PacBio sequencing data for the Coriell DNA sample NA12878 [29]. We used the high-confidence SV benchmark set distributed by Parikh et al. to evaluate the detection sensitivity of the different tools [30]. The benchmark set consisted of 2676 deletions and 68 insertions which were used to evaluate SV recall at two sequencing depths of 4X and 8X (Additional file 1: Fig. S4). For deletions, SVIM performed the best recall (0.79) at 4X depth, followed by NanoVar (0.71), Sniffles (0.58), and Picky (0.54). All the tools improved considerably at 8X depth but the same trend was observed. For insertions at 4X depth, NanoVar had the greatest recall (0.66), followed by SVIM (0.62), Sniffles (0.28), and Picky (0.22). At 8X depth, NanoVar and SVIM were tied for recall (0.82), while improvement was also seen in other tools. Similar to our simulations, NanoSV performed poorly in recall for both SV classes at both depths. Overall, NanoVar was observed to perform comparably well with SVIM on recalling deletions and insertions from real PacBio sequencing data. As the SVs in this benchmark set is non-exhaustive and could not be considered as the ground truth, the evaluation is incomprehensive and NanoVar would still require further testing on real experimentally validated SVs.

### Precise SV characterization in AML patients using NanoVar

**Fig. 3:**
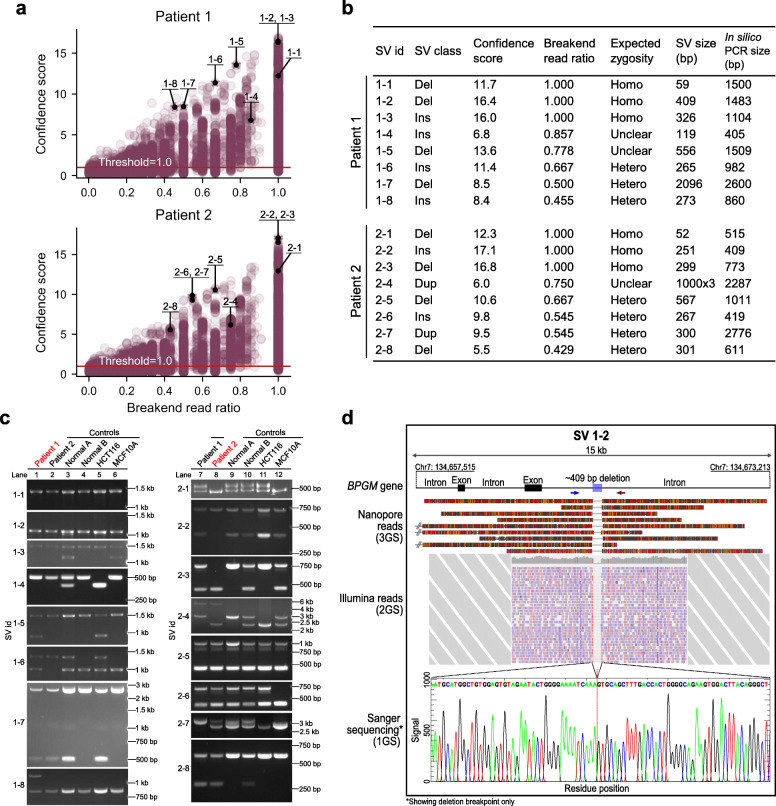
Precise patients’ SV characterization by NanoVar. **a** Scatter plots showing the confidence score and breakend read ratio of each SV characterized in patient 1 (top) and patient 2 (bottom). SVs selected for validations are labeled on the plots by their SV id. The red horizontal line indicates the confidence score threshold used for filtering. **b** Table displaying the details of SVs selected for validation for patient 1 and patient 2. The in silico PCR size refers to the expected size of the PCR amplicon without the SV. **c** Gel electrophoresis images of PCR products corresponding to each of the SVs in table **b**, amplified from the genomic DNA of patients 1 and 2, normal donors (normal A and normal B) and cell lines (HCT116 and MCF10A). Sample names in red (left image lane 1, right image lane 2) indicate the sample where the SV was initially detected. **d** Schematic illustrating a 409-bp deletion (SV 1-2) in the intronic region of the gene *BPGM* in patient 1, supported by 3GS nanopore reads (top), 2GS Illumina reads (middle), and 1GS Sanger sequencing chromatogram (bottom). Blue and red arrows represent the primer locations used for PCR amplification. For each nanopore read, base substitutions and base insertions are represented by red and orange markers respectively. Base deletions are represented by gaps. All nanopore reads have at least 90% alignment identity. Illumina paired-end short reads are represented by pink (forward) and blue (reverse) small rectangles, and the read coverages are displayed in gray above all the reads. The red dotted line on the sequencing chromatogram marks the precise breakpoint of the deletion at single nucleotide resolution

We tested the NanoVar workflow on two Asian patients diagnosed with acute myeloid leukemia (AML) (patient 1 and patient 2) to evaluate the workflow’s feasibility and SV characterization accuracy in low sequencing depth clinical samples. Genomic DNA extracted from bone marrow mononuclear cells of each patient was sequenced by nanopore sequencing using two to five MinION flowcells, generating about 12.4 Gb and 12.3 Gb of sequencing data respectively (Additional file 1: Table S3). The sequencing reads were applied to NanoVar for read mapping and SV characterization with a minimum support of two reads for each breakend to increase stringency for clinical data. NanoVar discovered a total of 14,215 SVs (3218 DUP, 6706 DEL, 2626 INS, 1348 BND, 317 INV) in patient 1 and 16,562 SVs (3019 DUP, 8727 DEL, 2576 INS, 1976 BND, 264 INV) in patient 2 (Additional file 1: Fig. S5). To evaluate NanoVar’s accuracy in these samples, we surveyed eight SVs from each patient for PCR validation. We selected SVs with a range of confidence scores and breakend read ratios which approximate SV zygosity (Fig. 3a). All the selected SVs are situated in autosomal chromosomes (Additional file 1: Table S4). Primer sequences were designed flanking the breakend location(s) of each SV according to the reference genome, and their referenced amplicon lengths (in silico PCR length without the SV) are recorded in Fig. 3b, along with their breakend read ratios and estimated SV sizes. PCR performed for each SV in their respective patient samples revealed that all 16 SVs were validated to be true based on their product size deviation (Fig. 3c lanes 1 and 8, Additional file 1: Table S5a). Moreover, the PCR results were agreeable with the SV class, SV size, and SV zygosities estimated by NanoVar, based on the number of PCR products (one product for homozygous, two products for heterozygous). All amplified products were gel extracted and their sequence identity validated by Sanger sequencing (Additional file 2). Besides PCR validation, all 16 SVs were also found to be supported by mapped 2GS short reads generated by Illumina WGS (Additional file 1: Fig. S6). Figure 3d illustrates an example of how a deletion SV (SV 1-2), characterized by 3GS nanopore long reads, can be supported by 2GS Illumina short reads and 1GS Sanger sequencing. The successful validation of SVs with varying breakend read ratios and varying confidence scores reinforces NanoVar’s precision in SV characterization in clinical samples.

### NanoVar recalls shared SVs validated by PCR

We went on to uncover shared SVs between patient 1 and patient 2 among the selected SVs by testing the SVs reciprocally in each patient by PCR using the same primers and cycle conditions (Fig. 3c lanes 7 and 2). Patient 1 was discovered to possess all eight SVs that were validated in patient 2, while patient 2 possesses six of the eight SVs validated in patient 1. In total, 14 out of 16 validated SVs were discovered to be shared between patient 1 and patient 2, leaving SVs 1-5 and 1-8 to be patient 1 specific (Fig. 3c, Additional file 1: Table S5). Next, we investigated if NanoVar was sensitive enough to capture these shared SVs in the respective patient. In patient 1, NanoVar was able to detect five out of eight shared SVs, while in patient 2, all six shared SVs were detected, aggregating to 11 out of 14 shared SVs recalled (Additional file 1: Table S5). We investigated the three undetected heterozygous shared SVs (SVs 2-2, 2-3, and 2-8) and discovered that they had insufficient or an absence of SV-associated reads at their loci, while still having reads originating from the “wild-type” allele. These results suggest that a higher depth of coverage (> 4X) would be required by NanoVar to confidently and broadly capture heterozygous SVs as observed in the simulated benchmarking.

### NanoVar characterizes polymorphic SVs

Upon assessing the presence of these SVs across more samples, we observed that most SVs appeared to be polymorphic. We tested the SVs in several samples consisting of normal hematopoietic stem cells (HSCs) from two non-AML Asian individuals (normal A and normal B), an epithelial colorectal carcinoma cell line (HCT116) and a Caucasian non-tumorigenic breast epithelial cell line (MCF10A). We found out that normal HSC samples do possess many of the SVs (13 out of the 16 SVs) which existed in at least one allele in either one or both HSC samples, while SVs 1-5, 1-8, and 2-4 were absent in both HSC samples (Fig. 3c lanes 3, 4, 9, and 10). Interestingly, these 13 shared SVs were also present in both patient 1 and patient 2, suggesting that these SVs might be prevalently found in cells, irrespective of AML disease. On the contrary, SVs absent in the HSC samples (SVs 1-5 and 1-8) were exclusively found in patient 1, except for SV 2-4, which was found in both patient samples. Out of these 13 shared SVs, 11 of them were also present in non-hematopoietic cell lines such as HCT116 and MCF10A (Fig. 3c lanes 5, 6, 11, and 12), leaving two SVs (SVs 2-7 and 2-8) undetected in either cell lines. Taken together, the majority of the SVs (11 out of 16 SVs) were commonly found across the samples regardless of AML disease status, cell type, and ethnicity. Moreover, most SVs exhibited different zygosities among the samples (all SVs excluding SV 1-1, 1-2, 1-8, and 2-5), which suggest a polymorphic nature. Within the scope of this study, we categorized the SVs into three groups based on their prevalence and zygosity variation across the samples: (1) rare sample-specific SVs (SV 1-8), (2) common SVs with no zygosity variation which might be due to an incorrect reference genome (SVs 1-1, 1-2, and 2-5), and (3) common SVs with zygosity variation or polymorphic SVs (remaining SVs) which constituted most of the SVs characterized by NanoVar.

### The NanoVar workflow is time efficient

We compared the CPU time and maximum resident set size (memory) used by the workflows of each tool for SV characterization in patient 1 to evaluate their processing speed and memory usage (Additional file 1: Table S6). Among 3GS SV callers, NanoVar stood out as the most time-efficient tool by requiring about tenfold lesser CPU hours than the rest to process 12 Gb of sequencing data using 24 threads. In real time, NanoVar took 196 min for the entire analysis of patient 1 which is the fastest among all other tools. In exchange for its speed, NanoVar employs about 1.7-fold more memory than the rest, having a higher memory cap of 32 GB.

## Discussion

NanoVar is a novel SV characterization tool that excels in accuracy and speed while overcoming the low-depth and error-prone sequencing of 3GS WGS. In simulated data, we showed that NanoVar outperformed existing 3GS SV callers by achieving high SV detection accuracies (*F*_1_ > 0.92) when using only 4–8X coverage datasets for homozygous and heterozygous SVs. NanoVar’s performance was reflected in low-depth real data such as sample NA12878 and clinical data where we successfully validated a small subset of SVs discovered by NanoVar (16/16) and demonstrated reliable estimations of SV class, size, and zygosity. Results from both simulated and real data also suggested that 4X sequencing depth may be suboptimal for comprehensive heterozygous SV discovery and would recommend a higher sequencing depth of 8X.

Although we propose the confidence score of 1.0 as a default threshold for running NanoVar, there might be situations where it is less effective and requires the re-training of the neural network model. The threshold score was observed as the optimal score for the balance of both precision and recall in simulated experiments. Thus, the effectiveness of the threshold is dependent on the resemblance of data characteristics between simulated and real environments, which may be quite different in certain situations. Such cases may include the usage of long reads from new sequencing technologies utterly different from ONT or PacBio, or analyzing genomes with vastly more proportion of repetitive sequences than the human genome. In these situations, the alignment profile of each read (e.g., mismatch errors, number of multi-maps) might be exclusive and thus requires re-training for a better fit using the tutorial provided on GitHub (see the “Availability of data and materials” section).

One major advantage of 3GS over 2GS SV calling approaches is the amount of raw sequencing data consumed. In our study, we showed that 12 Gb of 3GS data (4X coverage) produced a more comprehensive SV detection outcome than 160 Gb of 2GS data (53X coverage) when comparing the analysis done by NanoVar and 2GS SV callers (Fig. 2c). The considerable reduction in sequencing data requirements could speed up SV analysis and reduce computational resources. 3GS approaches may be used in large-scale SV-association studies or routine sequencing-based clinical investigations to analyze and store massive amounts of sequencing FASTQ/FASTA files more efficiently [5, 7].

Despite NanoVar’s high accuracy, many of its characterized SVs might be SV polymorphisms commonly found in the human population. We observed that most of the validated SVs found in our AML patients also existed with mixed zygosities in normal HSC samples and other cell lines, suggesting that they might be benign polymorphic SVs. As SV polymorphisms are widespread in the human genome [31–36], it is important to annotate these SVs by cross-referencing to collective polymorphic-SV databases to facilitate the discovery of disease-associated SVs. Alternatively, the GRCh38 human reference genome could be improved to encompass polymorphic sequence variations where polymorphic SVs could be readily identified [37]. The use of low-depth nanopore sequencing for accurate and routine SV characterization could supply a steady flow of knowledge to the construction of such cohort reference genome and inclusive SV databases.

## Conclusions

The NanoVar workflow is an accurate and cost-efficient SV characterization method that utilizes low-depth WGS nanopore sequencing data. As WGS approaches are arising as a potential clinical strategy, we hope that NanoVar will assist in the routine SV characterization in patients and the discovery of novel pathological SVs for precision therapeutics.

## Methods

### The NanoVar pipeline

NanoVar takes as input a WGS long-read FASTQ/FASTA file (at least 12 Gb) and a reference genome and outputs two VCF files (total SV and filtered SV) and an HTML summary report. The NanoVar workflow comprises of three main stages: (1) long-read sequence mapping, (2) SV characterization with read-depth calculation, and (3) artificial neural network (ANN) inferencing from a simulation-trained model.

#### Stage 1: Long-read sequence mapping

The first stage aligns long-read sequences to a user-provided reference genome using the tool HS-BLASTN [22] (version 0.0.5+). HS-BLASTN is an accelerated sequence alignment search tool that uses the MegaBLAST algorithm. We selected HS-BLASTN over other long-read aligner tools because of its faster computational speed and accurate read alignment, based on our evaluation. Before running HS-BLASTN, tools from NCBI-BLAST+ are used to build a blast database (makeblastdb [38], version 2.6.0+) and mask highly repetitive sequences (windowmasker [38, 39], version 2.6.0+). HS-BLASTN is run with the following parameters: “-reward 2 -penalty -3 -gapopen 0 -gapextend 4 -max_target_seqs 3 -outfmt 6.” The output is a BLAST-like tabular file containing alignment information of each read. Due to overlapping alignments within some reads, a Python script is used to trim the overlapped regions or select the best alignment based on alignment bitscore.

#### Stage 2: SV characterization and read-depth calculation

The alignment anchor sequences and divergent sequences/gaps of each read are analyzed by Python scripts to detect reads containing *novel adjacencies* (reads possessing split-read or hard-clipped alignments) and subsequently characterize their SV class. A *novel adjacency* is defined as two adjacent genomic coordinates in a sample genome that are not found to be adjacent in the reference genome. A novel adjacency is represented as two genomic coordinates in the reference genome, each known as a *breakend*. We use an algorithm of conditional control statements for novel adjacency detection and SV characterization, described in Additional file 1: Fig. S1. Any read that is found to possess a novel adjacency is labeled as an SV-associated read, otherwise, labeled as a normal read. Next, the read depth was calculated at each breakend for SV-associated reads and normal reads separately. This gives us the number of breakend-supporting reads *B* and breakend-opposing reads *O* at each breakend. Due to repetitive sequences in the genome, artificial breakends with unusually high *B* may be falsely detected. In order to filter out these untrue breakends, we define the upper limit of *B* as *U*, where breakends with *B* > *U* are considered outliers and removed. *U* is calculated by

$$ U=4\cdotp \frac{k}{n}\sum \limits_{i=1}^n\left|{x}_i-m(X)\right|+m(X) $$

where *n* is the total number of genomic locations chosen, *x*_*i*_ is the read depth at genomic location *i*, *m*(*X*) is the median read depth of all chosen genomic locations, and *k* is the constant scale factor 1.4826. The value of *U* is defined as four times the mean absolute deviation around the median (MAD) from the median in the distribution of a breakend read-depth assessment. This outlier detection method is an adaptation from Leys et al. where they proposed that the median absolute deviation is a more robust measure of dispersion than the standard deviation [40]. In our method, we use the MAD instead of the median absolute deviation to reduce fluctuations caused by discontinuous median integers. The breakend read-depth assessment is a sampling procedure to approximate the read depth of SV throughout the genome. It is performed by randomly choosing *n* number of genomic locations and calculating the number of reads covering each location after adjusting for *G*. This produces a distribution similar to a gamma distribution, and the median *m*(*X*) and MAD can be computed. According to our simulations, we empirically defined *U*, the deviation of more than four times the MAD from the median *m*(*X*), to be an outlier threshold, in the context of the human genome. Hence, any breakend which has *B* greater than *U* will be omitted and the remaining breakends will proceed to the next stage of ANN inferencing.

#### Stage 3: ANN inference

A trained ANN model is employed to improve SV characterization accuracy by evaluating read alignment characteristics and breakend read-depth information. For each novel adjacency, 23 scaled features are inferred by the ANN model which produces an inference value *P* ranging from 0 to 1. Next, *P* is exponentially scaled inversely according to the value of *B* and the final predicted score *S* is expressed logarithmically related to its error rate. *S* is described as

$$ S=-10{\log}_{10}\left(1-\left(\tanh (0.4B)\cdotp P\right)\right) $$

where *B* is the number of breakend-supporting reads at a novel adjacency and *P* is the ANN inference value of a novel adjacency. The hyperbolic tangent function is used to decrease the value of *P* non-linearly when *B* is low (*B* = [1, 2, 3]), as a low *B* confers low confidence. The value of *S* is proportional to the confidence level of a novel adjacency and is used to filter confident novel adjacencies from the total VCF output file to create the filtered VCF output file. A HTML summary report is also generated at the end of each run.

### Artificial neural network model and training

The features used by the ANN are described below (number in parentheses represent the number of neurons):

- Aligned/unaligned percentages flanking the novel adjacency (5)
- Alignment *E* values flanking the novel adjacency (2)
- Relative alignment bit scores flanking the novel adjacency (2)
- Alignment identities flanking the novel adjacency (2)
- The fraction of mismatches in alignments flanking the novel adjacency (2)
- The fraction of gaps in alignments flanking the novel adjacency (2)
- SV complexity—number of coexisting SV found at the novel adjacency (1)
- Total number of alignments found on read (1)
- Total number of SV that seemed to be captured by read (1)
- Number of different chromosomes the read aligns (1)
- The fraction of alignments less than 5% of read length (1)
- Number of breakend-supporting reads *B* (1)
- The fraction of breakend-supporting reads *B* over total read depth *B* + *O* (1)
- If SV is an insertion/deletion, the size of the inserted/deleted segment (1)

The value of each feature is scaled to the range of [0, 1] by min-max normalization. The Python library Keras [41] was used to build and infer the ANN model. The backend engine used with Keras is TensorFlow [42]. The neural network model is a feed-forward network consisting of a 23 neuron input layer, two hidden layers of 12 and 5 neurons sequentially, and a single neuron output layer. The rectified linear unit (ReLU) activation function is used for the two hidden layers, while the Sigmoid activation function is used for the output layer. Dropout regularizations were implemented after each hidden layer with probabilities of 0.4 and 0.3 sequentially. If *y*_*k*, *i*_ denotes the value of the *i*th neuron in the *k* layer, we have that

$$ {y}_{k,i}=F\ \left(\sum \limits_j{W}_{j,i}^k\ {y}_{k-1,j}\right) $$

where *F*(*x*) = max(*x*,0) denotes the ReLU non-linearity and ${W}_{j,i}^k$ is the neural weight between the *j*th neuron of the (*k* − 1)th layer and the *i*th neuron of the *k*th layer.

Ten million in silico 3GS reads simulated from a simulated genome consisting of 61,316 mixed zygosity SV were used to train a binary classifier ANN model through supervised learning. The ten million reads were distributed randomly into 20 sub-datasets before read-depth clustering to reduce the sequencing depth to 1X. The entire training dataset consists of 933,351 true and 41,186 false examples of novel adjacencies. Another simulated dataset (4X) with a different SV profile was used as the test dataset. Binary cross entropy was used as the loss function, and stochastic gradient descent (SGD) was used as the optimizer algorithm with their default parameters. The classification accuracy is collected and reported as a metric to assess the performance of the model. Sixty-three epochs were performed for the model training, with each epoch having 12,000 true and 12,000 false randomly selected examples and a batch size of 400 examples per iteration.

### SV genome simulation for test datasets

The template genome used for genome simulation consisted of the main nuclear chromosomes (chromosome 1 to Y) in the GRCh38 human reference genome assembly. The R Bioconductor package, RSVSim [43], was used to introduce novel adjacencies systematically in a reference genome to create different classes of SV. One SV simulation dataset consists of two genomes where one has 32,000 SVs of deletions (20,000), balanced inversions (1000), single tandem duplications (1000), and genomic sequence transpositions (10,000), and the other has 10,000 SVs containing only viral insertions which mimics novel insertions. The amounts for each SV were based approximately on SV occurrence reported by Chaisson et al. [44]. SV lengths were estimated by the *estimateSVSizes()* function in RSVSim, which takes reference from the Database of Genomic Variants (DGV) (Additional file 1: Fig. S2a). SVs were inclined to be positioned in repetitive regions using the “repeatBias” parameter in RSVSim. Viral sequences used for viral insertions were part of 54 viral genomes taken from GenBank [45] (Additional file 1: Table S7). The virus selection was based on their ability to integrate into the host genome. To simulate SV sequence variability, each novel adjacency has a 20-bp flanking region where bases had a 25% chance of single nucleotide polymorphism (SNP) and a 50% chance of introducing indels with a maximum indel length of 5 bp. A total of three datasets were generated consisting a total of six SV genomes. Their FASTA files can be downloaded from doi: 10.5281/zenodo.3569479.

### Mix zygosity SV genome simulation for the training dataset

The mix zygosity SV genome was created by three simulated genomes with varying number of SV from the same SV profile: genome A has 61,316 SVs (100%), genome B has 51,099 SVs (83%), and genome C has 30,659 SVs (50%). The SVs in genome C are a subset of SVs in genome B. Different numbers of in silico 3GS reads were generated for each genome: 2.5 million reads from genome A, 2.5 million reads from genome B, and 5 million reads from genome C. The combination of all the reads produced the simulation of homozygous SV (50%), heterozygous SV (33%), and low-confidence SV (17%). A homozygous SV only has breakend-supporting reads at their breakends while a heterozygous SV has both breakend-supporting and breakend-opposing reads at similar proportions. A low-confidence SV simulates a true SV event in low-depth circumstances and has a majority of its breakend reads being breakend-opposing.

### In silico third-generation sequencing (3GS)

*Nanosim* [46] was used to generate in silico 3GS reads from the simulated SV genomes. Read features, such as read length, SNP, and indel profile, were modeled according to that of real ONT MinION reads from patient 1 and patient 2, which are provided as input into *Nanosim*. 2,080,000 reads were generated for 4X depth, 4,160,000 reads for 8X depth and 6,240,000 reads for 12X depth datasets. Heterozygous SV datasets were created by having half of the reads generated from an SV genome and another half from the GRCh37 reference genome. Comparisons for read length and indel proportion between real reads and in silico generated reads are shown in Additional file 1: Fig. S2b and Fig. S2c. Statistics of reads and genome mapping can be found in Additional file 1: Table S8.

### In silico second-generation sequencing (2GS)

DWGSIM [47] was used to generate in silico 2GS reads from the simulated SV genomes. The generation of 2GS reads followed these settings: Illumina platform, 307-bp average insert size, 59-bp standard deviation of insert size, 150-bp read length, paired-end reads, 53X mean coverage across all regions, uniformly increasing per base error rate from 0.1% at the start of read to 1% at the end of read, and contains no mutations, indels, or random DNA reads. The insert size, read length, and coverage follow that of real whole-genome 2GS data of patient 1 and patient 2. Statistics of reads and genome mapping can be found in Additional file 1: Table S8.

### NA12878 benchmark sample

PacBio sequencing data and high-confidence SV truth set of the Coriell DNA sample NA12878 was retrieved from 1000 Genomes Project [29] and Parikh et al. [30] respectively. We downsized the original data generated by the Sanger Institute to 6.3 million reads and 12.53 million reads to archive 4X and 8X sequencing depth datasets respectively.

### Performance evaluation in simulation and real datasets

Each ground-truth SV breakend was treated as an individual true event, and thus, the size of the SV was implicitly considered. A 400-bp error distance from the coordinates of a ground-truth SV breakend was used to create an 800-bp ground-truth SV breakend region. For each tool we are testing, we extracted the breakend coordinates of all the SVs predicted in their VCF file. In order to justify a breakend match, the predicted breakend coordinate must fall within a ground-truth SV breakend region. This intersection was carried out by BEDTools [48]. For each simulation dataset, the SV callsets from the 32,000 SV genome and 10,000 SV genome were combined. Predicted SV breakends which fall near the N-gap junctions of the reference genome were manually filtered out as they were artifacts. Precision and recall were computed manually or by Scikit-learn (metrics.precision_recall_curve) [49], and *F*_1_ score was calculated by the equation: $F1\ \mathrm{score}=\frac{2\left(\mathrm{Recall}\times \mathrm{Precision}\right)}{\left(\mathrm{Recall}+\mathrm{Precision}\right)}$.

### DNA sample source

DNA samples used in this study were acquired from four individuals: two patients with AML (patient 1, patient 2) and two healthy donors (normal A, normal B). Patient 1 and patient 2 had the M5 AML classification (acute monocytic leukemia) with FLT-3 Asp835 mutations, but the absence of recurrent SV based on karyotyping. Patient 1 also has a mutation in the NPM1 gene. All subjects are of Asian ethnicity.

### Cell lines

The HCT116 and MCF10A cell lines were obtained from Horizon Discovery (HD PAR-007) and ATCC (ATCC CRL-10317™) respectively and grown in their respective recommended growth culture conditions.

### Genomic DNA extraction

Mononuclear cells (MNCs) of all individuals were isolated from the bone marrow. The bone marrow from the pelvic bone was used for patients 1 and 2, and the bone marrow from the femur was used for normal A and normal B. For patients 1 and 2, the bone marrow was diluted in phosphate-buffered saline (PBS) containing 2% HyClone™ fetal bovine serum (FBS) (GE Healthcare Life Sciences) and 2 mM EDTA. MNCs were then isolated by Ficoll-Paque layering using Ficoll-Paque PLUS (GE Healthcare Life Sciences) following the manufacturer’s protocol. For normal A and normal B, additional processing steps were carried out due to the presence of liquid fats. The femoral marrow was diluted in PBS containing 10% FBS, 3 mM EDTA, and 0.4% sodium citrate. Cells were strained using a 100-μm cell strainer and pelleted by centrifugation at 300*g* for 10 min at room temperature (RT) without acceleration and brakes. Red blood cells were lysed in 40 ml ACK lysis buffer (0.15 M NH_4_Cl, 1 mM KHCO_3_, 0.1 mM EDTA-Na_2_, pH adjusted to 7.2–7.4) at RT for 5 min. Cells were pelleted by centrifugation again with the same settings. The cell pellet was resuspended in PBS containing 2% FBS and 2 mM EDTA, and subsequently MNC isolation by Ficoll-Paque layering following the manufacturer’s protocol. MNCs of normal A and normal B were enriched for hematopoietic stem cells (HSCs) by CD34 cell surface marker selection using the CD34 MicroBead kit, human (Miltenyi Biotec) according to the manufacturer’s instructions. The buffer used for CD34+ cell selection is PBS containing 2% FBS and 2 mM EDTA. Genomic DNA of MNCs and CD34+ cells were extracted using AllPrep DNA/RNA/miRNA universal kit (Qiagen) and genomic DNA of HCT116 and MCF10A cells were extracted using the conventional phenol-chloroform extraction method.

### Nanopore whole-genome sequencing and base calling

High molecular weight genomic DNA (1–1.5 μg) was sheared to 6–10-kb fragments by the G-tube (Covaris). Library preparation was performed using ONT 1D or 2D Ligation Sequencing kits (SQK-LSK108, SQK-LSK208) following their protocol. FFPE DNA repair was not carried out. DNA ends were prepared using NEBNext Ultra II End Repair/dA-Tailing Module (New England Biolabs) for extended incubation time (30 min—20 °C, 30 min—65 °C). Ligation of sequencing adapters was performed using Blunt/TA Ligase Master Mix (New England Biolabs). Libraries were sequenced using the MinION sequencer on either R9.4 or R9.5 flowcells for 48 h without local base calling. Base calling was carried out by Metrichor or Albacore. Details of sequencing runs are documented in Additional file 1: Table S3. FASTQ/FASTA files were extracted from FAST5 files using h5dump (version 1.8.16) from HDF5 tools [50]. For the 2D protocol, the FASTQ/FASTA was extracted from the template strand instead of the combined strand if the complementary strand failed in quality.

### Nanopore read mapping and SV calling

Simulated data and NA12878 PacBio reads were mapped to GRCh37 genome assembly while patient samples were mapped to GRCh38 genome assembly. SV calling with NanoVar (version 1.2.7) was carried out with default settings with confidence score threshold at 1.0 for all simulated data and NA12878. For patient samples, “-f hg38” gap exclusion parameter and breakend minimum read coverage “-c 2” setting was used. For NanoSV [25] (version 1.1.6), Minimap2 (v2.17-r941) [51] was used with the “-ax map-ont” parameter for simulated data and “-ax map-pb” for NA12878. For patient samples, LAST (version 938) was used as the aligner instead with default parameters. The scoring parameters for LAST were generated from a 20,000 reads subsample using last-train. NanoSV was run with the default configuration parameters, and we input our own hg38 random BED file for coverage depth calculations. We called SV with Picky [24] (version 0.2.a) using the BASH script they provided. We used their recommended LAST parameters for read mapping: “-C2 -K2 -r1 -q3 -a2 -b1 -v -v.” Picky was run with default parameters as in the BASH script. For SV calling with Sniffles [19] (version 1.0.8), NGMLR (version 0.2.7) was used for read mapping with default parameters. Sniffles was run with the -s 2 parameter which allowed at least two reads as minimum support for an SV to be reported. All SAM file sorting, BAM conversion, and BAM indexing were carried out by SAMtools [52]. For calculating read mapping statistics, Minimap2 was used for read alignment and statistics were calculated using SAMtools.

### Illumina whole-genome sequencing, mapping, and SV calling

Genomic DNA (1 μg) was randomly sheared to 350-bp fragments with Covaris cracker (Covaris) followed by sequencing library preparation using the Truseq Nano DNA HT Library Prep kit (Illumina). Sequencing libraries were sequenced paired-end 150 bp on the HiSeq X Ten sequencing platform (Illumina) with the HiSeq X Ten Reagent Kit v2.5 (Illumina) to a mean depth of coverage of about 50x. Reads were mapped to GRCh38 genome assembly using BWA-0.7.17 [53] with the default BWA-MEM parameters and 24 threads. SAM files were processed to sorted and indexed BAM files using SAMtools [52]. 2GS simulated reads were mapped to GRCh37 genome assembly using Minimap2 with the “-ax sr” parameter. For SV calling with novoBreak [26] (version 1.1.3rc), sorted and indexed BAM files were input with default run parameters. A dummy BAM file was simulated (GRCh38) to be used as a matched normal control. The confidence score for each breakend was obtained from the QUAL scores in the output VCF file. For SV calling with Delly [27] (version 0.7.8), duplicated reads in the BAM files were identified by Picard MarkDuplicates [54] before running Delly with the provided hg38 exclude file and its default parameters.

### SV experimental validation

Polymerase chain reaction (PCR) was carried out to amplify SV-containing regions in the genomes of each sample. We used two different PCR master mixes. REDiant 2X PCR Master Mix (Axil Scientific) was used for conventional PCR amplification, whereas LongAmp Taq 2X Master Mix (New England Biolabs) was used for longer (> 1.5 kbp) or AT-rich PCR products. DMSO was added to a final concentration of 3% to increase the success rate of GC-rich product amplification. Primer sequences were designed using PrimerQuest Tool by Integrated DNA Technologies and shown in Additional file 1: Table S9. Forward and reverse primers were added to a final concentration of 0.4 μM each. Two to 5 ng of genomic DNA was used as the template in each 25 μl PCR reaction. Standard three-step PCR settings were used for most PCR reactions on a thermal cycler. Touchdown PCR conditions may be implemented for some reactions to reduce nonspecific products. PCR products were separated on 1% agarose TBE ethidium bromide gel by gel electrophoresis, and DNA bands were visualized by UV light. DNA fragments were excised and extracted using a cotton wool gel filtration protocol as described in [55] or QIAquick Gel Extraction Kit (Qiagen). DNA was subsequently purified using Agencourt AMPure XP beads (Beckman Coulter) following their protocol for PCR purification. Primary or nested PCR product sequences were validated by Sanger sequencing.

### CPU time and maximum memory consumption assessment

GNU Time (version 1.7) was used to assess the CPU time and maximum memory consumption of each tool. We assessed each tool by executing the following command: “/usr/bin/time -verbose -output=output.txt sh -c ‘Tool command’,” and the results are stored in the output.txt file. The CPU time is calculated by combining the user and system time, and the maximum resident set size is taken as the maximum memory consumption.

## Supplementary information

**Additional file 1.** Supplementary figures and tables cited in the main text.

**Additional file 2.** Sanger sequencing results of SV PCR validation for Patient 1 and Patient 2 samples.

**Additional file 3.** Review history.

## Data Availability

NanoVar is an open-source python package available on Bioconda (https://anaconda.org/bioconda/nanovar), Python Package Index (https://pypi.org/project/nanovar), and GitHub (https://github.com/benoukraflab/nanovar) [56], licensed under the GNU Public License v3. The source code can also be found on Zenodo (doi: 10.5281/zenodo.3569496) [57]. It operates on a Linux x86_64 operating system with Python 3.6 or above and requires the pre-installation of BEDTools. All simulation datasets and ground truths are available on Zenodo (doi: 10.5281/zenodo.3569479) [58]. The Coriell NA12878 sample PacBio sequencing dataset was downloaded from the 1000 Genomes Project (ftp://ftp.1000genomes.ebi.ac.uk/vol1/ftp/technical/working/20131209_na12878_pacbio/si/) [29]. The high-confidence SV benchmark set for NA12878 was downloaded from NCBI (ftp://ftp-trace.ncbi.nlm.nih.gov/giab/ftp/technical/svclassify_Manuscript/Supplementary_Information/) [30]. ONT and Illumina WGS datasets of patients 1 and 2 are deposited in and are available from the dbGaP database under dbGaP accession phs001847.v1.p1 (https://www.ncbi.nlm.nih.gov/projects/gap/cgi-bin/study.cgi?study_id=phs001847.v1.p1) [59]. Scripts used for data generation and analysis are available in the scripts directory on GitHub [56]. The datasets supporting the conclusions of this article are included within the article and its additional files (Additional files 1 and 2).
