# Supplementary material for: NanoVar: accurate characterization of patients’ genomic structural variants using low-depth nanopore sequencing
Source: Genome Biol. 2020 Mar 3;21:56. doi: 10.1186/s13059-020-01968-7 (PMC7055087; doi:10.1186/s13059-020-01968-7)
Supplement: Supplementary file 1 — Additional file 1. Supplementary figures and tables cited in the main text. [file 13059_2020_1968_MOESM1_ESM.pdf]

NanoVar: accurate characterization of patients' genomic structural variants using low-depth nanopore sequencing

Cheng Yong Tham, Roberto Tirado-Magallanes, Yufen Goh, Melissa J. Fullwood, Bryan T.H. Koh, Wilson Wang, Chin Hin Ng, Wee Joo Chng, Alexandre Thiery, Daniel G. Tenen, Touati Benoukraf

Additional file 1

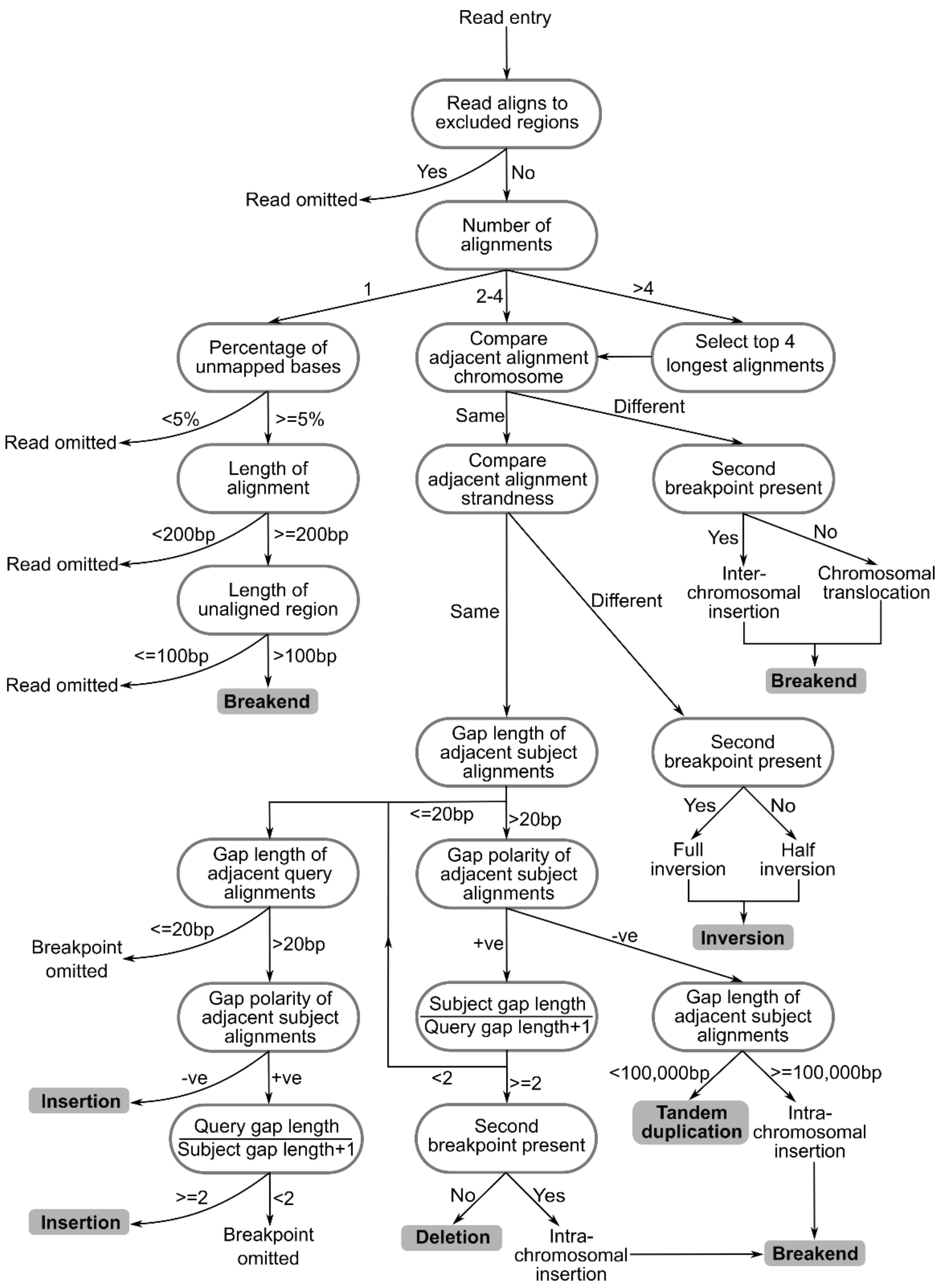

Figure S1: NanoVar SV characterization algorithm displayed as a decision tree. The algorithm consists of only conditional control statements coded in Python to analyse long-read alignment profiles for SV characterization.

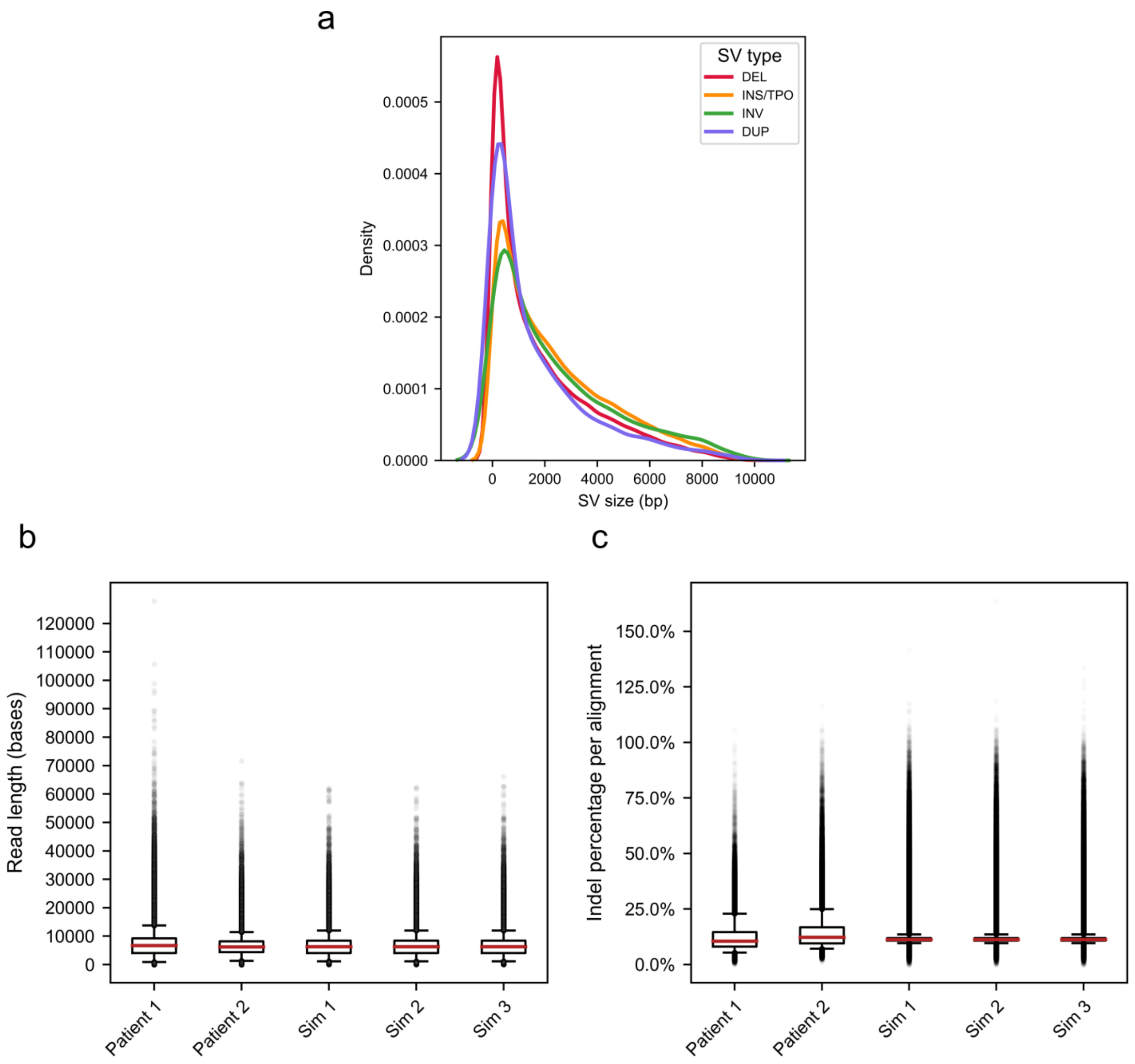

Figure S2: SV simulation and read simulation information. (**a**) Kernel density plot of the distribution of simulated SV sizes across the three simulated datasets. (**b**) Read length and (**c**) indel percentage distribution of simulated long reads and whole-genome patient Nanopore sequencing reads.

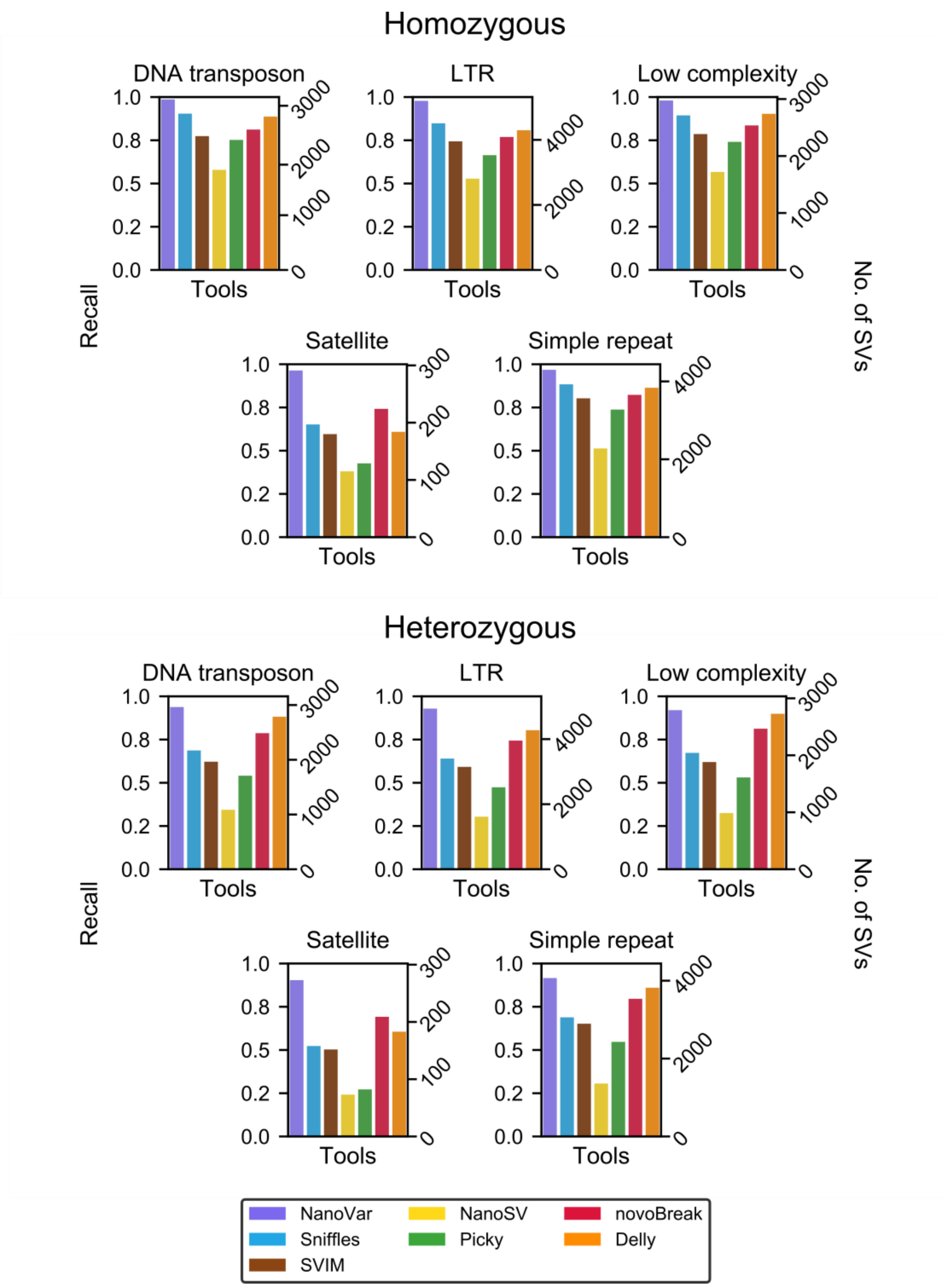

Figure S3: Repetitive sequence analysis of SVs recalled in homozygous (Top) and heterozygous (bottom) simulation datasets by the different tools.

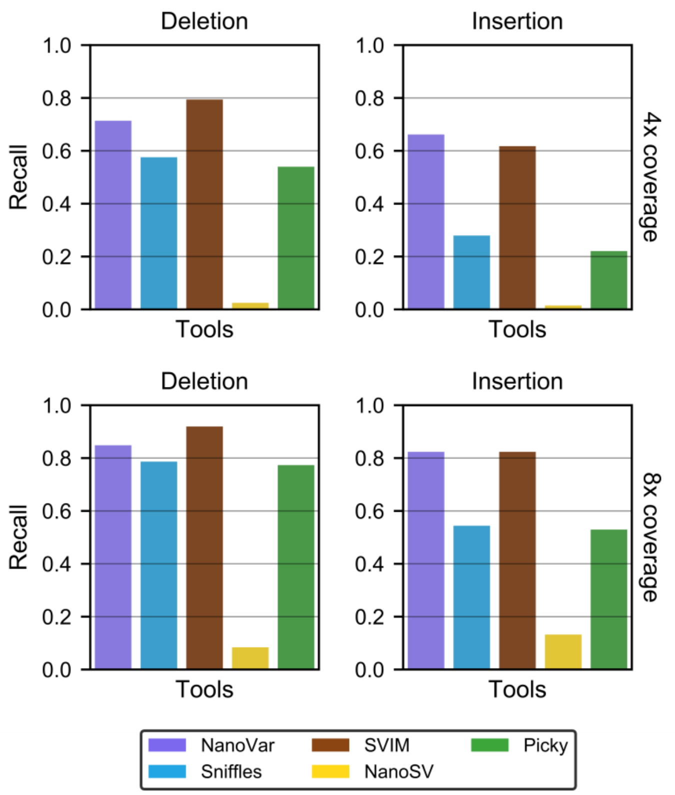

Figure S4: Recall benchmarking using NA12878 PacBio benchmark sample distributed by Parikh *et al.*, 2016 (2676 DEL and 68 INS). The number of reads used had been downsized to 4x coverage (Top) and 8x coverage (Bottom).

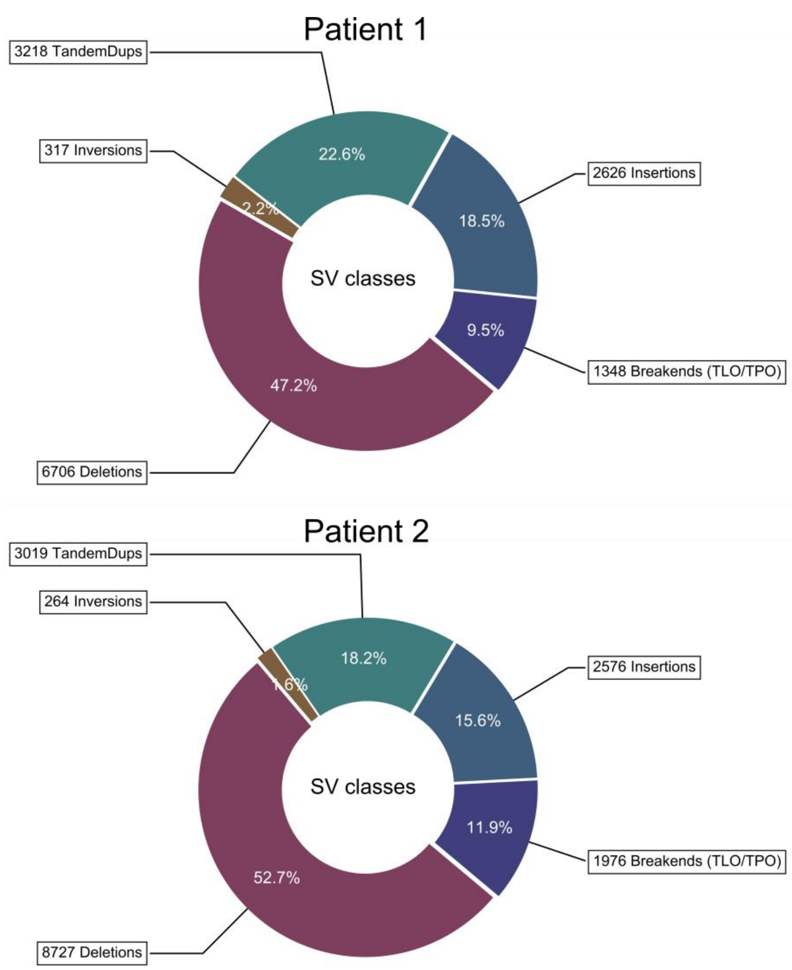

Figure S5: Donut charts showing the distribution of SV classes characterized by NanoVar in Patient 1 and Patient 2. TLO: Translocation, TPO: Transposition.

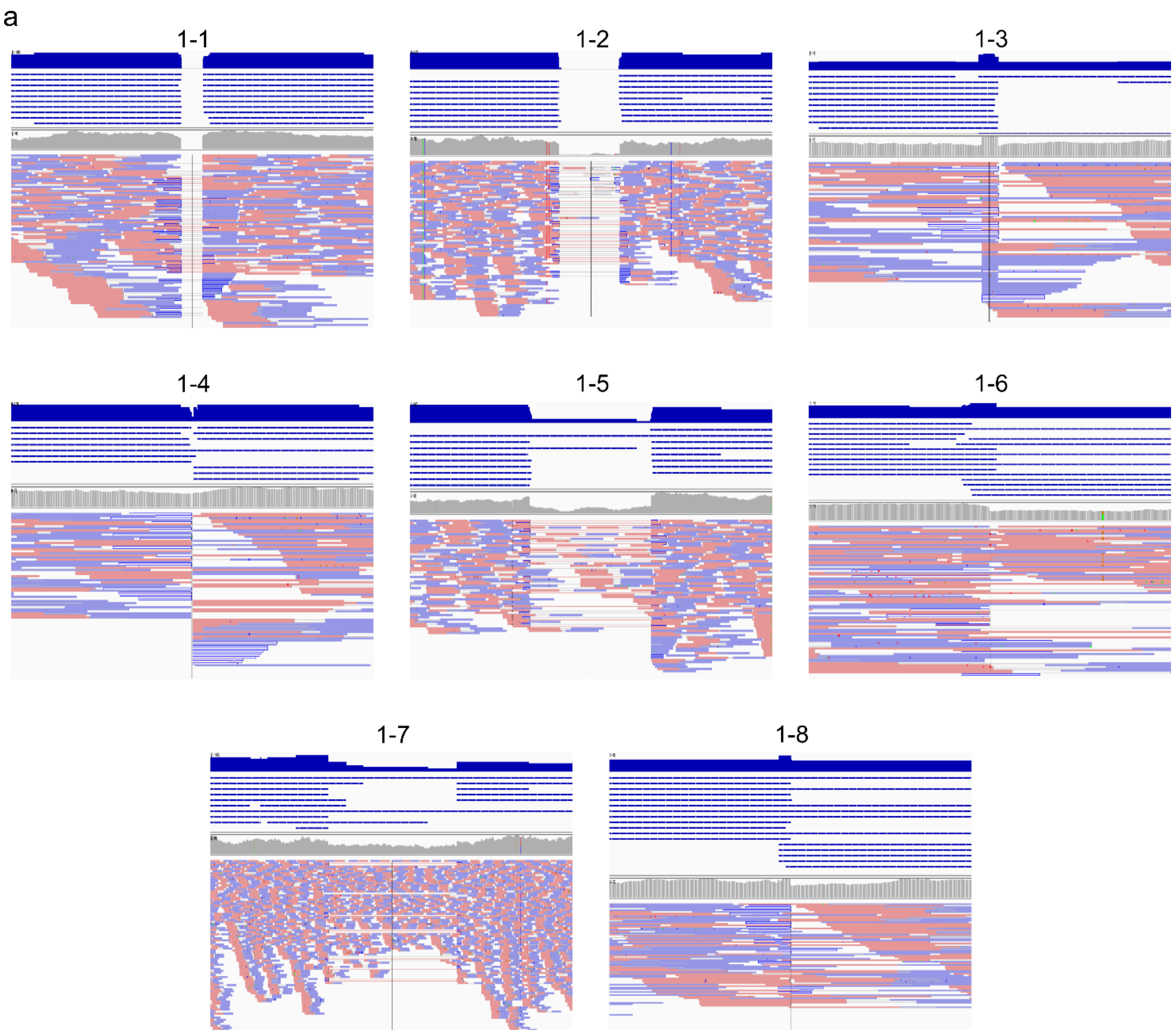
a
1-1
1-2
1-3
1-4
1-5
1-6
1-7
1-8

b

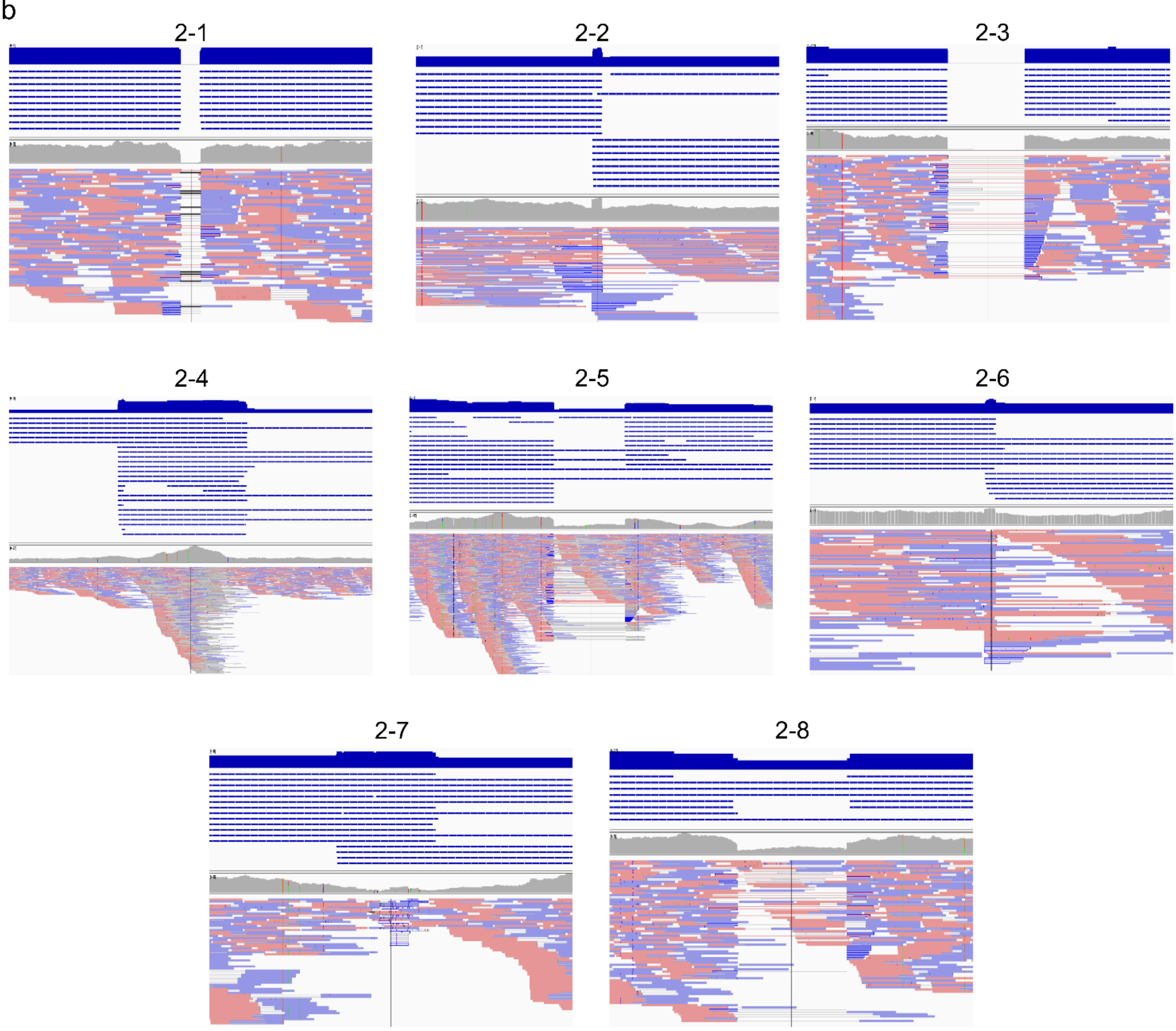

Figure S6: Genome browser snapshots of Nanopore long reads and Illumina short reads at each SV breakpoint location for SVs in (**a**) Patient 1 and (**b**) Patient 2. The top one-third of each snapshot displays the long reads in blue rectangles, while the bottom two-thirds displays short reads in red and blue rectangles. The cumulative read coverages for long reads (blue) and short reads (grey) are displayed above all the respective reads. The Integrative Genomics Viewer (IGV, https://software.broadinstitute.org/software/igv/) was used for the visualization of reads.

Homozygous

Tool	Optimum threshold score	Recall				Precision				F_1 score				Area under curve (AUC)			
		Sim 1	Sim 2	Sim 3	Avg	Sim 1	Sim 2	Sim 3	Avg	Sim 1	Sim 2	Sim 3	Avg	Sim 1	Sim 2	Sim 3	Avg
NanoVar	1.0	0.955	0.955	0.954	0.955	0.951	0.948	0.950	0.950	0.953	0.952	0.952	0.952	0.965	0.965	0.964	0.965
NanoSV	0	0.314	0.310	0.319	0.314	0.997	0.997	0.997	0.997	0.477	0.473	0.484	0.478	0.313	0.310	0.319	0.319
Sniffles	N/A	0.832	0.836	0.832	0.833	0.897	0.895	0.895	0.896	0.863	0.864	0.862	0.863	N/A	N/A	N/A	N/A
SVIM	0	0.791	0.790	0.791	0.791	0.719	0.720	0.714	0.718	0.753	0.753	0.751	0.753	0.759	0.758	0.759	0.772
Picky	N/A	0.586	0.583	0.583	0.584	0.023	0.023	0.023	0.023	0.044	0.044	0.044	0.044	N/A	N/A	N/A	N/A
novoBreak	27.5	0.616	0.630	0.609	0.618	0.965	0.949	0.963	0.959	0.752	0.757	0.746	0.752	0.654	0.666	0.649	0.650
Delly	N/A	0.708	0.707	0.706	0.707	0.977	0.975	0.977	0.976	0.821	0.820	0.820	0.820	N/A	N/A	N/A	N/A

Heterozygous (Sim1)

Tool	Optimum threshold score	Recall			Precision			F_1 score			Area under curve (AUC)		
		4x	8x	12x	4x	8x	12x	4x	8x	12x	4x	8x	12x
NanoVar	1.0	0.757	0.883	0.929	0.972	0.968	0.965	0.851	0.923	0.947	0.879	0.960	0.974
NanoSV	0	0.155	0.314	0.407	0.999	0.997	0.997	0.269	0.477	0.578	0.155	0.313	0.407
Sniffles	N/A	0.579	0.832	0.894	0.898	0.848	0.792	0.704	0.840	0.840	N/A	N/A	N/A
SVIM	0	0.641	0.793	0.848	0.674	0.571	0.483	0.657	0.663	0.616	0.586	0.742	0.804
Picky	N/A	0.384	0.600	0.666	0.015	0.012	0.011	0.029	0.023	0.021	N/A	N/A	N/A
		53x			53x			53x			53x		
novoBreak	27.5	0.585			0.977			0.732			0.625		
Delly	N/A	0.698			0.980			0.815			N/A		

Table S1: Precision and recall values of Figure 2a (Top) and 2b (Bottom). Sim # refers to simulation dataset #, where # refers to the dataset number. Avg: Average.

Homozygous

Tool	Optimum threshold score	DUP (2,000 breakends) Recall	TP	TP+FP	Precision	F_1 score	DEL (40,000 breakends) Recall	TP	TP+FP	Precision	F_1 score	INS (10,000 breakends) Recall	TP	TP+FP	Precision	F_1 score
NanoVar	1	0.938 (1875)	2038	2598	0.784	0.854	0.943 (37702)	41387	42551	0.973	0.957	0.929 (9290)	19312	27592	0.700	0.798
NanoSV	0	0.362 (724)	846	850	0.995	0.531	0.042 (1684)	1757	1758	0.999	0.081	0.135 (1347)	2694	2864	0.941	0.236
Sniffles	N/A	0.623 (1246)	1251	2154	0.581	0.601	0.852 (34083)	34400	35048	0.982	0.912	0.552 (5515)	7418	15346	0.483	0.515
SVIM	0	0.720 (1439)	1479	4635	0.319	0.442	0.764 (30554)	30981	42132	0.735	0.749	0.526 (5259)	10604	16616	0.638	0.577
Picky	N/A	0.612 (1223)	1223	1630	0.750	0.674	0.532 (21288)	29965	1412094	0.021	0.041	0.359 (3594)	6795	239574	0.028	0.053
novoBreak	27.5	0.429 (858)	986	1972	0.500	0.462	0.509 (20376)	24716	26215	0.943	0.661	0.000 (0)	0	0	0.000	0.000
Delly	N/A	0.703 (1406)	1407	2457	0.573	0.631	0.773 (30936)	31455	32570	0.966	0.859	0.001 (13)	13	40	0.325	0.000

Tool	BND (10,000 + 20,000 breakends) Recall	TP	TP+FP	Precision	F_1 score	INV (2,000 breakends) Recall	TP	TP+FP	Precision	F_1 score
NanoVar	0.822 (24673)	34826	36687	0.949	0.881	0.772 (1544)	1802	3123	0.577	0.660
NanoSV	0.467 (14024)	20971	24546	0.854	0.604	0.000 (0)	0	0	0.000	0.000
Sniffles	0.630 (18910)	23622	24669	0.958	0.760	0.741 (1481)	1511	2231	0.677	0.708
SVIM	0.544 (16331)	19205	26599	0.722	0.621	0.660 (1319)	1317	1884	0.699	0.679
Picky	0.377 (11319)	16973	17318	0.980	0.545	0.622 (1244)	1251	1268	0.987	0.763
novoBreak	0.776 (23279)	30766	32330	0.952	0.855	0.828 (1656)	3893	4056	0.960	0.889
Delly	0.863 (25889)	33992	34346	0.990	0.922	0.860 (1720)	3377	3534	0.956	0.905

Heterozygous (Sim1)

Tool	Optimum threshold score	DUP (2,000 breakends) Recall	TP	TP+FP	Precision	F_1 score	DEL (40,000 breakends) Recall	TP	TP+FP	Precision	F_1 score	INS (10,000 breakends) Recall	TP	TP+FP	Precision	F_1 score
NanoVar	1	0.774 (1548)	1626	1906	0.853	0.812	0.725 (29000)	30346	30920	0.981	0.834	0.618 (6176)	12512	17028	0.735	0.671
NanoSV	0	0.179 (358)	386	388	0.995	0.303	0.004 (151)	151	151	1.000	0.008	0.044 (440)	868	914	0.950	0.084
Sniffles	N/A	0.423 (846)	846	1428	0.592	0.494	0.549 (21976)	22100	22596	0.978	0.704	0.338 (3382)	4380	9152	0.479	0.396
SVIM	0	0.629 (1258)	1293	3278	0.394	0.485	0.614 (24570)	24848	35810	0.694	0.652	0.392 (3919)	3929	12642	0.311	0.347
Picky	N/A	0.406 (811)	810	1022	0.793	0.537	0.354 (14164)	19564	1412363	0.014	0.027	0.212 (2118)	3800	237349	0.016	0.030
novoBreak	27.5	0.356 (866)	908	1774	0.512	0.420	0.496 (19823)	22023	23366	0.943	0.650	0.000 (0)	0	0	0.000	0.000
Delly	N/A	0.690 (1380)	1380	2362	0.584	0.633	0.758 (30335)	30787	31891	0.965	0.849	0.001 (10)	10	30	0.333	0.000

Tool	BND (10,000 + 20,000 breakends) Recall	TP	TP+FP	Precision	F_1 score	INV (2,000 breakends) Recall	TP	TP+FP	Precision	F_1 score
NanoVar	0.579 (17377)	22049	22743	0.969	0.725	0.682 (1364)	1489	2046	0.728	0.704
NanoSV	0.242 (7262)	8691	10820	0.803	0.372	0.000 (0)	0	0	0.000	0.000
Sniffles	0.409 (12272)	14397	15180	0.948	0.572	0.534 (1068)	1073	1746	0.615	0.571
SVIM	0.383 (11503)	12936	20579	0.629	0.476	0.481 (962)	962	1550	0.621	0.542
Picky	0.181 (5420)	6771	6958	0.973	0.305	0.458 (916)	916	924	0.991	0.627
novoBreak	0.694 (20826)	25390	26296	0.966	0.808	0.818 (1636)	3419	3538	0.966	0.886
Delly	0.851 (25536)	33403	33594	0.994	0.917	0.864 (1728)	3378	3502	0.965	0.912

Table S2: Precision and recall values for different SV classes characterized by different tools presented in Figure 2c (Top) and 2d (Bottom). Numbers in parentheses in the recall columns represent the number of true SV breakends recalled. SV class annotation accuracy is considered in this analysis. For BND total breakends, 10,000 breakends are points of transposition location, and 20,000 breakends are the left and right coordinates of genomic sequences that were inserted. DUP: tandem duplication, DEL: deletion, INS: insertion, BND: breakend, INV: inversion, TP: True positive, FP: False positive.

	Flowcell chemistry	ONT protocol	Basecaller	Amount of gDNA used	Number of flowcells	Number of reads	Total number of reads	Total bases ($x10^6$)
Patient 1	R9.4	2D	Metrichor or Albacore	7.3 µg	2	441,637	1,793,667	12,434
	R9.5	1D	Albacore		3	1,352,030		
Patient 2	R9.5	1D	Albacore	2 µg	2	1,939,432	1,939,432	12,273

Table S3: Oxford Nanopore MinION sequencing details of Patient 1 and Patient 2

	SV id	SV class	Chromosome	Coordinates
Patient 1	1-1	Del	14	68528678-68528737
	1-2	Del	7	134665255-134665664
	1-3	Ins	18	40768187
	1-4	Ins	7	110844695
	1-5	Del	14	51911629-51912185
	1-6	Ins	14	53333449
	1-7	Del	4	79439473-79441569
	1-8	Ins	4	89221858
Patient 2	2-1	Del	4	142108392-142108444
	2-2	Ins	4	137377851
	2-3	Del	5	79717888-79718187
	2-4	Dup	4	7834853-7835893
	2-5	Del	21	10475720-10476287
	2-6	Ins	10	35706138
	2-7	Dup	6	66974314-66974585
	2-8	Del	3	194894010-194894311

Table S4: Genomic coordinates of SVs characterized in Patient 1 and Patient 2.

a

	Patient 1		Patient 2	
SV id	Validated	Shared-SV recalled	Validated	Shared-SV recalled
1-1	Y	n/a	n/a	Y
1-2	Y	n/a	n/a	Y
1-3	Y	n/a	n/a	Y
1-4	Y	n/a	n/a	Y
1-5	Y	n/a	n/a	Not shared
1-6	Y	n/a	n/a	Y
1-7	Y	n/a	n/a	Y
1-8	Y	n/a	n/a	Not shared
2-1	n/a	Y	Y	n/a
2-2	n/a	N	Y	n/a
2-3	n/a	N	Y	n/a
2-4	n/a	Y	Y	n/a
2-5	n/a	Y	Y	n/a
2-6	n/a	Y	Y	n/a
2-7	n/a	Y	Y	n/a
2-8	n/a	N	Y	n/a

b

	SV validated		Shared-SV recalled	
Sample	Individual	Total	Individual	Total
Patient 1	8/8	16/16	5/8	11/14
Patient 2	8/8		6/6	

Table S5: SV validation in AML patients and NanoVar recall status for PCR-discovered SVs. (**a**) Results of SV validation and shared-SV detectability by NanoVar in the respective patient samples. Y=Yes, N=No, n/a=Not applicable. (**b**) Summary results for Patient 1 and Patient 2.

Tool workflow*	CPU time (min)	Wall clock time (min)	Maximum resident set size (RAM in gigabytes)
NanoVar	1188	196	31.7
Picky	16910	1194	25.1
Sniffles	13187	561	18.9
SVIM	12795	558	18.9
NanoSV	32076	5278	21.5
Delly	28281	3374	33.2
novoBreak	57157	3519	45.0

*Comprise of sequence mapping and SV calling, using 24 threads

Table S6: Runtime and maximum memory usage consumed by the workflows of each tool using 24 threads for the SV characterization in Patient 1. For 3GS tools, 12 Gb of sequencing data was used, while for 2GS tools, 160 Gb of sequencing data was used. Data was collected using GNU Time. Please note that each tool workflow is inclusive of the read mapping step using their recommended aligner.

GenBank accession no.	Virus name
NC_012959.1	Human adenovirus 54
NC_001460.1	Human adenovirus A
NC_011203.1	Human adenovirus B1
NC_011202.1	Human adenovirus B2
NC_001405.1	Human adenovirus C
NC_010956.1	Human adenovirus D
NC_003977.2	Hepatitis B virus (strain ayw)
NC_001806.2	Human herpesvirus 1 strain 17
NC_001798.2	Human herpesvirus 2 strain HG52
NC_001348.1	Human herpesvirus 3
NC_007605.1	Human herpesvirus 4
NC_006273.2	Human herpesvirus 5 strain Merlin
NC_001716.2	Human herpesvirus 7
NC_009333.1	Human herpesvirus 8
NC_017994.1	Human papillomavirus type 136
NC_017996.1	Human papillomavirus type 140
NC_021483.1	Human papillomavirus type 154 isolate PV77
NC_033781.1	Human papillomavirus type 156 isolate GC01
NC_001526.4	Human papillomavirus type 16
NC_023891.1	Human papillomavirus type 178
NC_022095.1	Human papillomavirus type 179
NC_001357.1	Human papillomavirus 18
NC_001356.1	Human papillomavirus 1
NC_027528.1	Human papillomavirus type 201 isolate HPV201
NC_001352.1	Human papillomavirus 2
NC_001591.1	Human papillomavirus type 49
NC_001531.1	Human papillomavirus type 5

GenBank accession no.	Virus name
NC_034616.1	Human papillomavirus type 85 isolate 114B
NC_004500.1	Human papillomavirus type 92
NC_005134.2	Human papillomavirus type 96
NC_001596.1	Human papillomavirus type 9
NC_001401.2	Adeno-associated virus 2
NC_001729.1	Adeno-associated virus 3
NC_006152.1	Adeno-associated virus 5
NC_018102.1	MW polyomavirus
NC_020106.1	STL polyomavirus strain MA138
NC_020890.1	Human polyomavirus 12 strain hu1403
NC_024118.1	New Jersey polyomavirus-2013 isolate NJ-PyV-2013
NC_001538.1	BK polyomavirus
NC_001699.1	JC polyomavirus
NC_009238.1	KI polyomavirus Stockholm 60
NC_009539.1	WU Polyomavirus
NC_010277.2	Merkel cell polyomavirus isolate R17b
NC_014406.1	Human polyomavirus 6
NC_014407.1	Human polyomavirus 7
NC_014361.1	Trichodysplasia spinulosa-associated polyomavirus
NC_015150.1	Human polyomavirus 9
NC_001669.1	Simian virus 40
NC_022518.1	Human endogenous retrovirus K113
NC_001802.1	Human immunodeficiency virus 1
NC_001722.1	Human immunodeficiency virus 2
NC_001436.1	Human T-lymphotropic virus 1
NC_001488.1	Human T-lymphotropic virus 2
NC_001364.1	Simian foamy virus

Table S7: GenBank accession number and name of viruses used for SV insertion simulation to mimic viral insertion events.

3GS long-read

Sample	Number of reads	Read length (bp)		Total bases ($x10^6$)	Bases mapped ($x10^6$)	Percentage of bases mapped	Alignment error rate	Lengthwise genome coverage	Estimated sequencing depth
		Median	Interquartile range						
Simulation 1	2,080,000	6316	4370	13,304	12,198	91.69%	1.72E-01	90.55%	3.95x
Simulation 2	2,080,000	6322	4379	13,315	12,201	91.63%	1.72E-01	90.57%	3.95x
Simulation 3	2,080,000	6315	4374	13,300	12,190	91.66%	1.72E-01	90.56%	3.95x
Patient 1	1,793,667	6721	5226	12,434	11,308	90.94%	1.60E-01	91.19%	3.66x
Patient 2	1,939,432	6196	3797	12,273	11,191	91.18%	1.84E-01	91.01%	3.62x

2GS short-read

Sample	Number of reads	Read length (bp)	Total bases ($x10^6$)	Bases mapped ($x10^6$)	Percentage of bases mapped	Alignment error rate	Lengthwise genome coverage	Estimated sequencing depth
Simulation 1	1,090,215,422	150	163,532	163,433	99.94%	5.49E-03	92.05%	52.92x
Simulation 2	1,090,234,050	150	163,535	163,436	99.94%	5.49E-03	92.05%	52.92x
Simulation 3	1,090,321,944	150	163,548	163,449	99.94%	5.49E-03	92.06%	52.93x
Patient 1	1,084,425,108	150	162,664	160,274	98.53%	7.51E-03	94.52%	51.90x
Patient 2	1,096,966,824	150	164,545	162,251	98.61%	7.23E-03	94.51%	52.54x

Table S8: Statistics of 3GS and 2GS reads and read mapping of real reads and simulated reads. All reads were mapped using Minimap2 aligner except for 2GS real reads which was aligned by BWA-MEM. Simulated reads were aligned to GRCh37 reference genome while real reads were aligned to GRCh38 reference genome. Statistics were calculated by SAMTools. The sequencing depth was estimated from the Lander/Waterman equation.

SV id	Forward primer (5'-3')	Reverse primer (5'-3')
1-1	GCTCCAGTCAGATTGCACTAA	GCCTCCATAGACCTACAGTTTC
1-2	GAAAGGGAAGGTGATGCTGATA	AGCCTGTTTATGGTAATAGGGATAG
1-3	AGAACATTATTCCACCAGCCA	AGTGAGAAGAAGCAGAGAAATCA
1-4	GCTTGTAACAAACGGATGGG	CCTCTTAATTGCACTTTGACTCC
1-5	CAAAGTGTAAAGTGGCTTGGTG	GCTGGTAACCTTGTAGGGAATAG
1-6	GAGGCACAGACACATCCAAA	CTGCTTCAGCTTCTCTGTTCA
1-7	CAACCTCCTTGGTTAAATGTGTTC	CAGGATTAGTTGCTTGTTTCCTTAC
1-8	ATATGCCAGTCTGTGCTGTG	GCCCTGCCTATGCCTTA
2-1	AGAGACATCTCCAAACCTACAA	TCTGGGAAATCTGGCTCTAC
2-2	CATGGCTGTAAGTGGGTTTATC	CATATCGTTAGATTTCTTCACTGCT
2-3	CCCTAACTTCCAGGCTTCTA	AGTCACCTATGATCCTGCTTAT
2-4	CAAGGCAGAACCTCCAATG	ATCCTTGTGCCAGGTTATCT
2-4	*AGAGAGATCCAAGCTGTAGTC	*TTTGCATACACTGTGAAGTAGG
2-4	CAAGGCAGAACCTCCAATG	*GGCAGGTTTATATTTGTTTCAGTTC
2-5	GAGGCACAGGGACCATAAC	TGCCACAACAAATGGTTTACAA
2-6	AGTTGTAGCATCTACGTTAGACA	TGGGCATGAAGAGTGATAGG
2-7	GGGAATACTCTGAGATGTGTGA	CTGTATATCACTAGGATGTAGTAAAGTG
2-8	GCGTTGAGTTTCACAATGACTAA	GGGAGTTCTTTCACCTCTTTCT

*Nested primers for reamplification of PCR product

Table S9: Primers used for SV validation in Patient 1 and Patient 2 shown in Figure 3, and nested primers used for PCR product reamplification for Sanger sequencing (SV 2-4).
