# Supplementary material for: NanoVar: accurate characterization of patients’ genomic structural variants using low-depth nanopore sequencing
Source: Genome Biol. 2020 Mar 3;21:56. doi: 10.1186/s13059-020-01968-7 (PMC7055087; doi:10.1186/s13059-020-01968-7)
Supplement: Supplementary file 2 — Additional file 2. Sanger sequencing results of SV PCR validation for Patient 1 and Patient 2 samples. [file 13059_2020_1968_MOESM2_ESM.pdf]

Additional file 2

This Additional file contains Sanger sequencing results of SV PCR validation in Patient 1 and Patient 2. Results are presented in FASTA format where the read id corresponds to SV id.

Note: If two PCR products were observed in the case of a heterozygous SV, the products were sequenced separately and differentially labeled as "top" for the top band and "bottom" for the bottom band when resolved on an agarose gel (Refer to Figure 3c). Do note that these sequences were used to verify the sequence identity of the PCR products and may not span or elucidate the SV breakpoint junctions.

>1-1

NNNGNNNNTCANCTGTTGCTAAAGCCATGAGANAGGCTGGCACCAACCAGTAAAGAATTCC
CTCCAGTCTTCCGTACCATACATCCTGAATGGAGCATAATCCCTGCATGGTTTCTAGCCCAT
CATAACCTTGCTACACTGTTTCATAGAAAAACTTCAGCCATTGAGAATGATGTCATTCATCATA
TTTCCTCTTTCTTTGCCTTTTGCTGCCAGGAAGCCTTAAACAAATTTAATACTCAATTACAGTA
ATAATTAACTCAGATTTTGGGCATAATTATTTTCCCTTCTTCCTCTATGGTTTAATTTTCCCTTT
ATTATTCAACCAACTTATTGCATATGTGACTTTCATTGTAAACTACCTCAAGTACACTTTAAAA
GTTGGACATAAATCATAAAGTATAAATAAATTAAGTTTTATCTGATCTCTTGTAAAAACCCATG
ATCAGCCTGTTAAAATTCATATTACTAAACATCTTGACCAGTAAATGTCAGGAGGGTGGCATG
AAGAGCAAGAAAGAGAAAGCTCTGAGTCAAGGTCCATATTTAGTGGGAAAAAAAATCCTGGC
AAATATAGATTTGTTCAGAACTAATTATTTACCTTTTAAAATGGGTTACTTAATACTTATTCTAG
TATGTCCTGTGAGCATCTGTTGGGTGTCAGGCACAGTTTTAAATGTTATTTCATTAATTTTGTG
AGGTAGGTATTATCATTTCTAGCTGAAGAATAAGAAAACTAAGAATCAAACAAGTTAGATAAC
TTGCTCCCAGTCTCTCAGTTAGTAAGTGATAAGTCTGCAGAAGAAACCCTGGTCCGTCCAAC
TCCAAGGAATTCACAACCCTATTCTGTTTCATCAAATACAGGTTTTCTTTCTCTAGGTGGGTTA
TGTTGAAATGGTGAACCAGGCTGAAGCCACAGGTTGACTGAGTTCTAAATCCAGAAGCACAG
CTAGAGGCTCCTGTGGAATGAGAGGGACTAAAATTGCTTCAAGTGCAGTGGATTACTGGGG
TTAGACTCACTTCTAAAAATGAAACACTGAAGTGGGGCTTTCTTTTTTTCTTTGTTTTTGAGAC
AGGATCTCACTCTGTCACCCAGGCTGGAGTGCAGTGGCACAATCTTGGCTCACTGCAACTTC
CTCCTCCTAGGTTCAGGCAATTCTCCCACATCAGCCTCCCCAGCAGCTGGGACTACAGGCG
CGTGCCACCACGCCAGGCTAATTTTTGTATTTTTTGGTACAGACAGGGTTTCGCCATGTTGG

>1-2

NNNNNNNNNNNNNNGANTTGAATAACTCTGTTATGTGAAGAAGCCAGCCACAAGGGATCACAT
ATTAGAGGATTTCATTTATATGAAATACCCAGAATAGGCAAATCTTTAGAAACAGAAGGTAGA
GTGGTTGTTGCCTAGAGGGTAGGGAGGATGGAAGATTTTGCAAGTGATTTGCTGATGGGTA
CAGAGTTTCTTGTTGGGGTGATAAAAATATTCTAAAATTGATTGTAGTGAGGGTTGCACAACT
CTGAATATGCTAAGAGCCATTGAGTTGTACACTTTGTGTGGGTGAATTGTATGTGAATTATAT
CTCATTAAAGCTGGAAAAATAATCAATGGAATTGAAAGGTTAAAGGATTTGTCATTGGCCTAG
AAAACAGTTGGGGGTGGCAAACATATGGAAGTGATAACAAAACTGAAATGCATGGCTGTGGA
GTGTAGAATACTGGGGAAAATCAAAGTGCAGCTTTGACCACTGGGGCAGAAGTGGACTTAC
AGGGCTCATTGGGAAATAAGCAGGCATTGGTCTTCAGGCAGCTTCCAGTCCTTCATCTAAAG

GGCTTTTTATTTTATTTTATTTTTTATTTTTATTTTTTTAAGTTGGAAGTATGAAACTGAATGACC
TTTTCTTTTCTTTTAGAGATTAATGACATTTTCTTTTCTTTTCTTTTCTTTTCTTTTTTAGACAAA
GTCTCGCTGTGTTGCCCAGGCTGGAATGCAGTGGTTCGATCTTGGCCCACTGCAACCTTCG
CCTCCTGAGTTCAAGCGATTCTCATGCCTCAGCCTTCTGAGTAGCTAGGATTATAGGCGTGT
ACCACCACACCTGGCTAATTTTTGTAATTTTTTAGTAGAGATGGGGATTNGCCATGTTTGCCA
GGTAGNTCTCAANTCCTGNGTCTCAAGCGATATATTCACTTCAACCTCTCAANTCTGAGAATA
CGTNNNGATCCNCTGCTCTCCTGGCTATAAAGAATTTTTAAATTTAGCCTTACATTTTTAATNN
NATCCTNTTACCTTAACNTNNAANTGTTTANNCCTTNNAANNN

>1-3

NNNNNNTCNNANATAATAACTGGAGTGTAGCCATTTTTAGGTGGAGGCATTAAGGAAAAGAT
TGGAGAACCACGTAATTATGGTGACAGAGGAAAGAAGATGAAGAAAGAGAATTCTGATTTTA
GCCTGCAGTAATAGAAGTGTAACACCTATAAGAAGAAGAACAACTCTCCTAGTTTGCTCACAT
AGGTGAGATCACATTGGTTGCATTAATTGCAAGGCACTATGCTCATTTCCAATCTCTACAATA
TCATAAACCATGCCTGGTAGTCTTTTGGGGGCCTATTCATCATAGCTGTAAAAAAATCACTTT
CTAATGGTTAAGCACATATGTTACCTGCCAACGAATGTCTCAAAGGTTAGAATTAGACACACC
ACGGAATCTGAGACTTACTACCACATTTGATTGCAGCTTGTGATGCCAATGCTTGTGTGTGG
CAGCGACTTAATACACTGCTCTCTTTTTTTTTTTTTTTTTTTTTTTTTTAAAAAGGGNACTCCCCC
ANNNCCCCNNGTGGGGANCNGTNGGGCCGATCCTGACANNCNAGAAAGCCCCCCCCCCGG
GGNNCCCCTTTTTCTNNNACNTACACCAGAATAATCGGGTNCNAGGTACCCTCCCNCCNNC
CGCGTAAATTTTGNNTTTTTCNNNAAAACGGGTTTTCACCGGGATTAACACTAGGNNCCTCT
CCCNAGNNCTGCANNATNTACCCT

>1-4

NNNTNCNAGNNTTTGCCTTCATCCTATGGTTAATCAATAGGGGAGCTTTCAGATGTATTCAAG
CCAGATGACACTGTAGTTTAGAAAGATAAATCTAGTCCTACGCAGAAGACAGAGTGGGAGAT
AAAGCACTTAGTGGCAGGGAAGACAGTAGCGCACCAGCATGGCACATGTATACATATGTAAC
TAACCTGCACAATGTGCACATGTACCCTAAAACTTAGAGTATAATAAAAAAAA

>1-5_bottom

CNTTTGCATAGCTTATAATACCCTATGTACATGTGTATGTATGTGTGTGCTTGGGGTGGGAAA
ATATGAATAAATTCTGTTACGGGGTAGGGAAGGGAATGGTTATCAAGGCCTATGAGAATGAA
ATAGTCCCTAACCAGTAACTTGGAGTTCTTGTGTCCTGACTTGGGAAGCCACAGAGTTGAAT
GGCCAAAGATGTGACCTTGGAGACCCCTTGGCTGAGTTTTTTCGTGGCCCCTCCACTTACTG
ACTGGGAAATCTTGTAAATTTCCCGTACCACATCTTAACCTCTCATTCCTCATCAGGGCAATG
AGAACTTAACGACCTTTTGACCTTTTCTCTTATTATCGGCCGCAGAAGGACCCGGCCGCAGC
ATGAAGAAGATAAATCCCACTATGAAGTCAGCGCCCAGGACCACCTTCTTTTTTTTTCGGTTT
C

>1-5_top

ANNNNNNTTACGNCGAAATAGCCCTAAAAATCCCCTATGGTACATGGTGGTATGTATGGTGT
GTGCTTGGGGTGGGAAAATATGAATAAATTCTGTTACGGGGTAGGGAAGGGAATG

>1-6_bottom

NNNCNACATGTGTCTTTTCTTTTCTGGTCCAGGACTTTATCCAGGAACACACATTGAATACAG
TTGTCATGTCCCATCACTCTCCATCGATCTGGAATAGTTCTTCTGTCTTAAAGATGACAGGCC
TTCACTATATATGCAGCATGTCCCTCAACCTGAGTCTGTTTGCTGTTTCCACATGACCAGACT
CAGGTCATGCATTCTTAGCAGGAGTACCACAGAAATGATTCTGTGCTCATCTGGAGAAGTCA
GCATTTTCCCTCTGCATTTGATGAATATTTTGTGGCAGAATTGCCCAATATTATAAATAATATA
CTGTCTCTATGCAAATCTGTGTCCAGCTTCCATTGGTGACTCCTGTCTATATCAACTACCAAA
TGGTGCCAGATGGAGATTATCTATTCCCATCATTTGATCTACATTGATTAATTGGCATTATACT
GTAAATAAATGGTTTCCTTTCTACCTCATTTCTTTATTTACTCATTTATTTCTATCAGTGCAATG
TTATGGTTTTCTATTTTGCTTCATGAATTATAATTAGACATGCTAATTTATTTTGATGCACAAAT
TGTCCTAGATTTGGCCAGTGTGAACTCCCTAAACCTGGCTTCTGTGTCCCTTTGACAGGTCC
CTATCATTCCTTAAGCATTTCCTACTTCCTAGCATAACAACACATTCTAGGCTTACCTTATACT
TTCCCTGCCCCGAGCCTGTAAGCAGCCATTTCTCCAGAAGTCCTTGTTCCTAGCAGTGGAAG
ATGGTATTTAAAAACATAGGTCTGGATGGGATGCTAGATGTGCTTATTACTATTGGGGTGTTA
CTGCTCCTAGACACTCTCAGTGGATAGGACAAGCTCTCTGTGTAGACATACATACACATACA
TACAAATATATACTCACACACAAACATATATTCTATTAGAGTTGAACAGAGAAGCTGAAGCAG
ACCCN

>1-6_top

NNNNNGNNNCCTAACAATGGTGTCTTTTCATTTTCTGGTCCAGGACTTTATCCAGGAACACA
CATTGAATACAGTTGTCATGTCCCATCACTCTCCATCGATCTGGAATAGTTCTTCTGTCTTAA
AGATGACAGGCCTTCACTATATATGCAGCATGTCCCTCAACCTGAGTCTGTTTGCTGTTTCCA
CATGACCAGACTCAGGTCATGCATTCTTAGCAGGAGTACCACAGAAATGATTCTGTGCTCAT
CTGGAGAAGTCAGCATTTTCCCTCTGCATTTGATGAATATTTTGTGGCAGAATTGCCCAATAT
TATAAATAATATACTGTCTCTATGCAAATCTGTGTCCAGCTTCCATTGGTGACTCCTGTCTATA
TCAACTACCAAATGGTGCCAGATGGAGATTATCTATTCCCATCATTTGATCTACATTGATTAAT
TGGCATTATACTGTAAATAAATGGTTTCCTTTCTACCTCATTTCTTTTTTTTTTTTTTTTTTTTTT
TTTGAANGNGGTTTTCCCTTTGCCCCCCGGGGGGGGGGGAAGGGGGGGAATCCCAACCCC
GGGAAACCCCCCCCCCCGGGGTTCCCCCCTTTCCCCGGCCCCCCCCCCCGGGGGAGGGG
GGACAACGGGGCCCCCCCCCCANGCCCGNNNATTTTTTTTTTTTTTAGAAAAAAGGGGTTTT
CCCCGGGTTACCCGGGGGGGCCCCAACCCCCGGACCCCGGGACCCCCCCGCTCCGCCCC
CCCAAAGGGGGGGGATAAAGGGGGGGACCCTCGGCCCCGGGCNNTTCTNNNTTTNGNAAN
NACCNNTTTNNTTTTCGNGCGGGNCNNGTGTGGGNGTCNATATTTTNNNNACAAAAATATNN
GNGGACNGTGAGNNNTTTTNNGAGNNCAATGNNNCCCANANNTGGNNAGGNCAAGCTCCTT
AATAAGGGNNNNNGGCCTCCCTAAAAANNNCCCNTATCTACGTTNGGATTTTCTAGGGTNCC
TAGTAATATTNTTATC

>1-7_bottom

AGTTTGCTTACTTATATGTAGCTATTGTAATGGGATTGTTTTCCAGATTTTTTCAGATAATTTGT
TATTAGCATAAAAGAGGTGAGATTCACTTAATATTGTGTTTTGGGGAGAAATGTACTATTAAAT
TATCATCTGTTCTCTCAAAACCAAGTGAAGAATTAAATG

>1-7_top

NNNNTTNNNNNNNNNNNATGGTNGCTTNNNGNTAANNGNGGATNGGTTTTCCCAGAATTTTTT
CAGAATAATTTAGTTATTAGTNGTATAGAAATGCTACTGATTTTTGTATGTTGATTCTGTATAG
TGATACTTTCCTAAGTTACCTCATTAGTTATAAGTCTTTTATTTTTAATTATTTTATTTTATTTAT

TTTCTTATTTTTACTTTTTTTTTATTTGTATAGAAATGGTTTTCTGTTCCTGTGCTAGTTCACTTA
GGATAATGGCCCCCAGCTCTGCCCATATTGCTGCAAAGGACATAATCTCATTCTTTTTTATGG
CTGCATAGTATTCCACGATGAATATGTACCACATTTCCTTTAACCAGTCTACCACTGATGGAC
ATTTAGGTTGATTCCATGTCTTTGCTATTGTAAGTAGTTAATAGTAGTTTCTTATGATTCTTTGT
ATTTATGTGGTATCAATTGTAATGTCTCTTCCTTCATTAATGATTTTATTTATTTAAGTGTTCGC
TCTTTTTTTTCTTAGTTGGTCTATCTAAAGTTTTCTCAATTTTGTTTATTTTTTCAAAAACGTACA
CTTGGTTTCTTTGATGTTTTGTGTTGCTTTTCTAGCCTCTATTTTATTTATTTCTGCTCTGAAAG
TTAGTATTTTCTTCCTTTTGCTAAATTTGGGCTTAGTTTGTTCTTTTTCTCATTATCCGAGGTGT
AAAGTTAGGTTGTTTGAGATTTTTCTTCTTTCTTAATGTAGGAATGTAGGTATTTATCACTGTA
TCTTCCCCCTTAGAGTTGCTTTTGTTACAGTTCATAATTTTGGTATATTGTGTTTTCATTTTTCT
TTTTATAAATATGCTTTTTGATTTCTCTTTTGATTTCTTCTTTGACCCATTGATTGTGTGGATAT
AGGTTGTCTAATTTTCCCTTATTTTTGAATTATCCAAGTTTCTTCATGTGATTGACTTCTAGTTT
GGTACCAATGTGGCCTAAAATATGTCTGATGTNNT

>1-8_bottom

NNNNNNCCCGCTTTTATCTTTACATCAAAGAATGGAAGTTGGCCCTAGCTCTTTCATTTAAAA
AAAA

>1-8_top

AAATAGTCCACCGTTTTTATCTTTACATCAAAGAATGGAAGTTGGCCCTAGCTCTTTCATTTAA
AAAAAATCAGAAAAACAAACACTATATGGTATCACTTATATAGGGAACCTAAAAAAGGTAAAC
ACATAGAAATAGAAAGTAGCATGGTGTTTGCCAGGGGTAAGGGAGTGGATTT

>2-1

NNNNNANNGNCTGTGGGTTATAAACGGTACCAAAAAGTAACACATTTTACCTTTCCTTTTTAA
ATAGATTAGAAAATAATTTCCTAAGGAAAGAGTCTGTCTCATTTTTCAAGCTACAATTCACAAT
GGTGATTTCAAACATATTCCAATCTCCACTGGCCCTGATTTCACTAATCGCTTCTAACACAAC
AGTCTCTGTGCATGGACATAATTGATATTAGCTTCCCTATCCTACTCCACACCTCTGTGGCAG
GGCTACATCCCTGTATGTGTTTTAAAAAACTTTTCTTACTATCTATCCAACCATCCATTTCACT
CGTGGCAGCTGAGGGAGGGGACCAGAAGACCAGCACTGGACACTGGGCTGCACGCAGTCT
TGCCCACACTATCAACTGCTGTAGGCACATGTAGAGCCAGATTTCCCAGAATAACCANNAGG
CCCGCACTAATACNCNTTCCCAACTGTTGNTCAGCCTGAATGT

>2-2

TNNNNNGGNNAAGAAAGCATTCTGTACTCTAGCAGTCAAGAATATGCTACTCTCAAATCTCT
GACTATG

>2-3

NNNACNTNNNNACGGATCCTTATTCTTTCCATCCACAATGATGTTGAATGGCCTTTAGTATAT
CTACAAAATGAACACTTTCTATTACGGCATAATTATGCTGTGTAACAATTTATTCATTCATGGT
TTCATGAAATTTTTTCTTAACTCCCTAATGGCATAAAAGTTCCTTTTAACCTAAGCTATTTTATA
TTATAGTTTGCTGAACTGTTATTCAGCAATGCATATTTTTTTCAGCTAAACACATTAATATTAAA

TTAATTCTTGGTGAAATATTAATTGTAGAGTTAATGATTGCTTTTAAGAATTCCATTTTTCAAAA
GNNCCATACAAATGACTACCACCATTNNTCTCCTCAACCCATNAGAAACCCCTGTCTCAGCC
ATGCANCTTCCCGATCGATGNTGNNGNTTTGACNNNTGGTTNNCCCATNNGTAGCTACCNAN
GNATGCATATNNCCNNNNNGACATTCTTNNCGTTAAATCTCNCTGANCATCCCTGAAGNNTA
TCCTCATGTNTGACTTCTNNTCTCTGANNTCTGATCATTGATCATTANGCTTCCGCCCNCGNN
NCNCNCGTCTGNNCATATT

>2-4_bottom

NNNNNCANAGNNGNCNGGCAAATACCCATAAGCAGGATGGAGACCCCAACGGAATGTAACT
TGGAGTTAAGAGTGAACTGAAACAAATATAAACCTGCCTTTGAATGGACTGCGGGTGGCCTT
AAGGTTACCTGGGTGGCTCTTGAATGGACTGCGGGCAGCCTTAAGGTTACCTGGGTGGCTC
TTGAATGGACTGCGGGCTGCCTTAAAGTTACCTGGGTGGCTCTTGAATGGACTGCGGGCTG
CCTTACAGTTACCTGGGTGGCTCCTGAATGGACTGTGGGCTGCCTTAAGGTTACCTGGGTG
GCTCTTGAATGGACTGCGGGCTGCCTTAAGGTTACCTGGGTGGCTCTTGAATGGACTGCGG
GCGGCCTTAAGGTTACCTGGGTGGCTCTTGAATGGACTGCGGGCTGCCTTAAGGTTACCTG
GGTGGCTCTTGAATGGACTGCGGGCTGCCTTAAGGTTACCTGGGTGGCTCTTGAATGGACT
GCGGGCTGCCTTAAGGTTACCTGGGTGGCTCTTGAATGGACTGTGGGCTGCCTTAAGGTTA
CCTGGGTGGCTCTTGAATGGACTGCGGGCTGCCTCAAGGTTACCTGGGTGGCTCTTGAATG
GACTGTGGGCTGTCTTGAGGTTACCTGGGTGGCTCTTGAATGGACTGCGGGCTGCCTTACA
GTTACCTGGGTGGCTCTTGAATGGACTGAGGGCTGCCTTAAGGTTACCTGGGTGGCTCTTG
AATGGACTGCGGGCTGCCTTAAGGTTACCTGGGTGGCTCTTGAATGGACTGCGGGCTGCCT
CAAGGTTACCTGGATGGCTCTTGAATGGACTGCGGGCTGCCTAAAAGTTACCTGGGGGGCT
TTNGAATGGACT

>2-4_top

GTAGTGGGGAANAGCTGTAGTCAGGGCATGCCAAAGAGGGGGTCCCTGGCAAAATACCCAT
AAGCAGGATGGAGACCCCAACGGAATGTAACTTGGAGTTAAGAGTGAACTGAAACAAATATA
AACCATGCCTNA

>2-5_bottom

NANNNANNNTAGCAGGATGTGTATATACGTATATTAGAAATACTGAAGTTCAGTATTCTGTTA
CATGAATTTCTTTGATATTAGGCATTTCTGTATTATACTTCTTTTTTTATTTACTGTTGCTTCTA
CATATTCTTCACATCGGGGACTGGCGGTAAAATAAGCTGCATTTTCCTAGCTGTGATTGTGAT
GTAAGCAAGATGCATGATGCAAGACAAAACATTTTGAGAAATACAAAAACAGCAGTTCCAGA
ATTATTGGTGAAATTAAAATACCAAAAGAATTGAAGATCGACAAACTAAGACTGTGGCTGTGC
TTTCACAATTTTTAAGGACTGAAGTAGAAATATTCTTTTACTGTTGATCATGAAACACATTTTAA
TGCACATAATTGTCAATCATTTNNCAGTGGCAAAG

>2-5_top

NNNNNNNNNTANNTTAGCAGGATGTGTATATACGTATATTAGAAATACTGAAGTTCAGTATTC
TGTTACATGAATTTCTTTGATATTAGGCATTTCTGTATTATACTGCTTTTTTTATTTACTGTTGC
TTCTACATATTCTTCACATCGGGGACTGGCGGTAAAATAAGCTGCATTTTCCTAGCTGTGATT
GTGATGTAAGCAAGATTCTTTGGTTTCCAGCTATTTTTTTGTTAAAGTCACTGGCATTTTCATA
TTTTGTTGAACATTACCAATTCTTTGGTCTTTACCAGTACAGTAAATACTAACCAAAGAAATGT

AGATTTACTTTAAGTTCTGTTTCATGGTGGGCAAATTATTAAGTTGATTTTAATGATTTTCTATA
ATAGTATTATATGAGCTTTTCTAGGAATTCACTTCAAATTTAAACGAACATTTTAATAGGAAAC
ATCTGAAAGATCTAGTTTGTGATAGCTAGTGATTGGATTATCAGATGTTATCTGGAGGTACAA
GCTAGCCAACCAGTAGAACGGTGAGCCAGTGCTGTCTAATAGAAATATGATGCAGACCATAT
GTGTAATTTTAAATTTTCTAGTAGTTACACTTAAAAAAAGGTGAAATAAATTTTAATGTATTTTA
TTTAACCTGGTATATTCAAAACAATATTTCAGCATTTAGTATAAAAATGAACAAGATGCTTTAT
ACTTAAAACATCAGTTCTGGCTAGCTGCATTAGAAGTGCTCGGTGGTCACATATGATGGTGG
CTGCCTTATTGCACGATGCAAGACAAAACATTTTGAGAAATACAATAACAGCAGTTCCAGCAA
TTATTGCTGATATTAGAATACCAAAAGAATTCACGATCGACAAACTNNCACTGTGGGTGCNCT
TTCACAACTGTTAANNGACTGACGTAGCACCTATTGATTTNCCGTTGAACATCGAACTCCATT
TAANGGCNGATACTTGTTAGCNTTTTCGTTGCCGACAATTCTCGATN

>2-6_bottom

NATGCTCTTGTATTTTTCTAGAGAGCATGAAGTTATTGTTTTTTTCTACATTTAATACGTATAAC
TTCAAAAGAGACCAGAAAAGAATAGAAATAAAATTGAATCTCTCTATAGTCTGAGATGAAATG
AAATAGTTGTGGAGAATACGAAAGACGGACGAGACAACGGGGCCACGCCTTCTCCGGTGT
GCAGCGGATCGTTTGAGTTCACTTGTCCGTGTTTATCGGGGCGCATCCTCTCCTGTTCTTCC
TTGCTTGTTCTGGCATTCCTTCACCTAAGCTAAGCTGCCAAGAAGCCCAACTCCCTGCCGGC
CCCTCCGTTCGTTTGGGACTTCCGTCTCGTCGACATGAAACCGTGTAGCGCAGAACCCAAA
CCGTCTTTTCATTTTCATTCAGACTATCGATTGGATGATTTATCTTAATATCA

>2-6_top

CNNNNNNTCTTGTATTTTTCTAGAGAGCATGAAGTTATTGTTTTTTTCTACATTTAATACGTAT
AACTTCAAAAGAGACCAGAAAAGAATA

>2-7_bottom

NNNANAGNNAGNTGAGTGTTTGATAGGGTACACCTAGGGGGGTAAACCTTCCTGTCTTAAAG
AAT

>2-7_top

NNANGNTGATGATGAGTGTTGATAGGGTAACACCTAGGGGGGTAAACCTTCCTGTCTTAAAG
AATTTTATTTTGAGCATCTATAGAAAACTGCTTGAAGCAAGTCAAGTGAAATTTAGTTTTCTAA
TTCAGACTTGCAAATAAAATGCAAATTTGAGAACAAAAGAAATGAAATACTGCACACTTTAAAA
TGAACTGTGAATTGTAATATTTCTGAATAGGCCTTCTCCTAATATACACTAATTCTCTTTAGTG
TCTTTGATTAACATTACCGCCTCTGTCCCCTTACTTCTCGCACCCCTTGCAGTCAAAGAAAAG
CCTTGGAGTGTTGCTAGAAGAAAGAAATAATTTGTTATTGACTGATGAGACGCGCCTAGAAAT
GACTAATTATTATGAAACCTGCGAGGGAGGGTTACCTGCATTCACGATTTTTTAAGGCCGAG
TGGAGGGGGCGTGCAGTTTATTTTTCCTTTCTCGATAAACTAACCTTGCCGGTTTACATGATA
AATCTAAAGTGGGGGTCTCGAATTAGGATCGATATCTTTGTTCAACTGAACCTCGACCACCTA
ATTCAGGATAATGGATCTGCTTTATGAAGTGACCCTGATCCTTATATTCGCCTTTCGCCATTT
GAGTATGCGTAGGTAGTCTGAAATCTGGTTCCATGTTCACAATGCTAGCCACTGCTCGTCTG
TAG

>2-8_bottom

NNNNTCNCNGNTNGATCNATATTCTTTTCTTTCTCAGCTTTCTCAATCATTTTGGTGAAACAA
GTTCTTTCTCTCCTTTTCTAAGAAAGAGCGCATAGTAAGTAAATTTTTGTTCCTGTCCTGACAC
AACTGAAGAAGTTTTTAGTTTATTCTCATACTTAATTGATGTGGAATTTTAGGTGGAAAAATAT
CATCTCTCAATTTTGAAAGCACTGTGCCATTGTCATGTTGTTTCCAACTTAGTCATTGTGAAAC
TCAACGCA

>2-8_top

GCNTTCTCTGATTGATCCATATTCTTTTCTTTCTCAGCTTTCTCAATCATTTTGGTGAAACAAG
TTCTTTCTCTCCTTTTCTAAGAAAGAGCGCATAGGCCGGGCGCGGTGGCTCACGCCTGTAAT
CCCAGCACTTTGGGAGGCTGAGGCGGGCGGATCACGAGGTCAGGAGATCGAGACCATCCC
GGCTAAAACGGTGAAACCCCGTCTCTACTAAAAATACAAAAAATTAGCCGGGCGTAGTGGCG
GGCGCCTGTAGTCCCAGCTACTTGGGAGGCTGAGGCAGGAGAATGGCGTGAACCTGGGAG
GCACAGCTTGCAGTGAGCCGAGATCCCGCCTCTGCACTCCAGCCTGGGCGACAGAGCGAG
ACTCCGTCTCAAAAAAA
