# Supplementary material for: NanoVar: accurate characterization of patients’ genomic structural variants using low-depth nanopore sequencing
Source: Genome Biol. 2020 Mar 3;21:56. doi: 10.1186/s13059-020-01968-7 (PMC7055087; doi:10.1186/s13059-020-01968-7)
Supplement: Supplementary file 3 — Additional file 3. Review history. [file 13059_2020_1968_MOESM3_ESM.docx]

**1^st^ round**

**Reviewer 1**

The authors present a new algorithm named NanoVar that was built to align and call structural variants using data generated on Oxford Nanopore devices. This is much needed, as long-read aligners and variant callers are not yet adequate, and NanoVar looks promising. The authors were thorough in their manuscript, and it is well written. There are several points, many of which are practical in nature, that I believe will strengthen the manuscript. There are a few concerns as well.

Minor:

1. The authors lump insertions and translocations into a class they call "breakends". After the initial NanoVar run, could they take all "breakends" and perform a second alignment using sequence within the SV to determine whether it is a translocation, and where it came from? Or, more simply, consider secondary alignments from BLAST? I do not think this is critical for publication, but I do think it would simplify results if possible.

2. "with an increased in NanoVar's recall" -> should be "increase"

3. It's unclear to me whether the in silico PCR length in Figure 3b is with or without the SV. The text doesn't appear to specify: "Primer sequences were designed flanking the breakend location(s) of each SV according to the reference genome, and their referenced amplicon lengths (in silico PCR length)…" I assumed that it must be the predicted length without the SV until I looked at SV 1-2. The product size is much closer to the predicted size minus the SV size. Can you clarify this in the text and figure legend?

4. I believe the following section header is inaccurate because, unless I misunderstood, NanoVar actually discovered the SVs and they were validated by PCR: "NanoVar recalls SVs discovered by PCR."

5. Should be "possess" (or remove "do"): "We found out that normal HSC samples do possessed…"

Major:

1. The authors make an important point in their paper that I feel contradicts one of their previous conclusions. The authors first state that coverage >4x does not meaningfully impact NanoVars results: "These results imply that 4X is the minimal coverage required for optimal NanoVar SV characterization and that increasing the coverage above 4X will not affect its performance."Based on Supplementary Figure 3b, I would generally agree, but the authors then point out that 3x-5x coverage may be insufficient to identify heterozygous SVs: "We investigated the four undetected shared-SVs and discovered that three of them (SVs 2-2, 2-3, 1-7) were in fact captured by NanoVar but did not pass the confidence score threshold of 2.6, possibly due to insufficient read depth coverage (3X to 5X) for heterozygous SV detection. The other undetected heterozygous shared-SV (SV 2-8) was not detected due to the absence of SV associated reads at its locus. This SV locus was found to be under-sequenced, covered only by two reads that likely originated from the wild-type allele in Patient 1. These results suggest that a higher depth of coverage (>4X) would be required by NanoVar to confidently and broadly capture heterozygous SVs." In my opinion, the authors need to be careful about advertising 4x as sufficient in the abstract and other text. They should clarify that 4x is sufficient to identify homozygous mutations, and whether that applies at the "clinical" threshold of 2.6, or the "standard" 1.4. These details are important for end users. This should also be clarified in the discussion.

2. I have a concern about masking repetitive sequences in the BLAST database. My understanding is that repetitive sequences were historically masked because short-read technologies are inherently incapable of handling them. Part of the promise of long-read technologies is the ability to resolve many of these regions. I think this is biologically important because there are many repeat expansion diseases (e.g., Huntington's, ALS/FTD, spinocerebellar ataxias, etc.). There is even a repeat expansion implicated in Alzheimer's disease. Would repeat masking prevent NanoVar from identifying those repeats? I believe it would. e.g., if you look at the 'GGGGCC' repeat from C9orf72 in hg38, you will see that it is in lower-case, which I believe BLAST will treat as masked.

3. It's not clear to me how "genomic location" was determined when benchmarking NanoVar to other algorithms. I believe the section titled "Performance evaluation in simulation datasets" is where this is briefly described. How much error was allowed for mutations to be called the same? i.e., how much error in the breakpoint location and size? This is an important consideration in SV calling. Perhaps the other callers identified the "same" mutations, but with less precision on the location?

4. I see the authors provide source code and great documentation for NanoVar on github. It is important to provide all scripts and data used to generate all data. I see the simulated datasets, but it is not obvious to me where I could find scripts to rerun all analyses.

5. I do not see an explanation for how the two confidence thresholds, 1.4 and 2.6, were determined, which is important for readers to know. More importantly, however, is whether those confidence scores are ubiquitous across situations, or are they a result of training on the neural network? i.e., can I simply download NanoVar and rely on those thresholds, or will I need to define new thresholds?

6. Do users need to re-train the neural network in certain situations? It would be very helpful for end users if this were briefly explained in the discussion, and particularly on github. In either case, authors should provide a brief example on github for how to train, and assess whether the training is successful.

**Reviewer 2**

The authors discuss NanoVar, a structural variant caller specifically tailored to low-coverage long-read sequencing, which is very relevant considering high sequencing costs on these platforms. I am confident that the field of long read SV discovery hasn't saturated yet. I noticed that the authors mention the definition of SVs to be larger than 50 bp in their background section but include smaller variants in their simulations, and to remove those in later evaluations since this size range is highly inaccurate. It might be a better idea to be consistent here. The background section mentions that structural variants account for most of the genetic variation in the human population, which requires to be nuanced, as it accounts for the most genetic variation in terms of nucleotides, but not in the number of variants, which would be SNVs.

The authors compare the performance of their tool to other tools for SV discovery from long read sequencing using simulated data. While simulating data has its merits and provides an attractive simplification of the real world, I get the impression that the simulation used to compare the SV calling algorithms is not representative of what can be expected from actual structural variants in the human genome. The variant length and number of variants distribution used for the simulations (supplementary figure 2a) is not representative of what is seen in real human SV data, which relatively show many more smaller variants (50-100 bp) and fewer large variants (> 10 kb) (Audano et al., 2019; Chaisson et al., 2019; Zook et al., 2019). It is also not clear to me if these SVs are simulated in both heterozygous and homozygous states. I also believe the random locations of SVs are inappropriate, as real SVs are significantly more frequently found in repetitive regions. This probably explains why the short-read sequencing algorithms perform relatively well in your comparison but are never this precise in any real data. Given that outdated base calls were used to provide read features for the simulation of reads these are unlikely to represent reads which are obtained using current base-calling versions. Due to these limitations, I recommend the authors to also do their comparison using real variant data, for example from the GIAB sequencing and SV identification study (Zook et al., 2019), by downsampling a deeply long-read sequenced genome or by simulating reads from a human genome assembly. I would also encourage the authors to use a specifically-tailored algorithm for comparing their truth and test sets in precision-recall assessment, such as truvari (https://github.com/spiralgenetics/truvari). It is not entirely clear how you used bedtools and scipy for these calculations.

It seems to me that the number of SVs identified in the patients (7000 and 7761) is much lower than the number of SVs which is expected from human genome sequencing. Could the authors comment on this discrepancy? Could the masking of repetitive regions in the HS-BLASTN step or the low coverage sequencing explain this? I wonder if 4x coverage is overly restrictive, and the performance of these tools would be much better at 8-10x coverage, while still, affordable considering increasing throughput on ONT MinION and especially PromethION. It surprises me that increasing the throughput above 4x has a neglectible impact on the performance of NanoVar (Supplementary Figure 3), and I wonder if this would be replicated with real data. I see the authors remark that a higher coverage could be necessary for heterozygous SVs (page 12, line 43), which are probably the most frequent anyway.

I failed to install your software on both a Ubuntu 19.04 and a CentOS7.7. On Ubuntu the subprocess32 installation failed (_posixsubprocess.c:16:10: fatal error: Python.h: No such file or directory #include "Python.h" compilation terminated. error: command 'x86_64-linux-gnu-gcc' failed with exit status 1). I see you use Python2.7 during the installation, although it is very much recommended to move everything to Python3 as Python2 is getting deprecated by the end of this year. My CentOS7 turned out to be incompatible due to an ldd version < 2.16. It may not always be trivial or possible for users to update such components. I recommend the authors to make their tool available through bioconda, as this offers a very convenient way of installing the software in a reproducible environment on various operating systems, also for users less comfortable using the command-line.

Furthermore, I have some minor comments:

-The patient genomes were base called using albacore, suggesting that more recent basecalling using guppy could further improve results, as substantial improvements have been made to its accuracy. I wholeheartedly understand that it's impossible to keep up with the speed of software developments, but it could be interesting to see how the results are with a modern basecaller.

-It might be interesting to include the SV caller SVIM (Heller and Vingron, 2019) in the comparison, which we have seen is also rather sensitive, and probably has a higher value of being included compared to Picky which has a prohibitively high false-positive rate.

-I wonder if using Sniffles with -s 2 is overly restrictive for this dataset, as it can be expected that some structural variants will only be supported by a single read in such low coverage data.

-The supplementary table 8 mentions alignment with bwa mem, which is appropriate but not optimal and the authors might want to consider replacing the aligner with minimap2, but as this is not the aligner used for the variant calling this wouldn't affect any performance results.

-While the authors did not mention this, I assume your tool is equally well applicable to PacBio SMRT sequencing?

-Finally, you might find the SV caller comparison in our paper on SVs in PromethION sequencing interesting (De Coster et al., 2019).

I came across three typos:

-Page 6 line 31: simulated dataset -> simulated datasets

-Page 9 line 9: an increased -> an increase

-Page 13 line 6: do possessed -> do possess

Best regards,

Wouter De Coster

Audano, P.A., Sulovari, A., Graves-Lindsay, T.A., Cantsilieris, S., Sorensen, M., Welch, A.E., Dougherty, M.L., Nelson, B.J., Shah, A., Dutcher, S.K., Warren, W.C., Magrini, V., McGrath, S.D., Li, Y.I., Wilson, R.K., Eichler, E.E., 2019. Characterizing the Major Structural Variant Alleles of the Human Genome. Cell.

Chaisson, M.J.P., Sanders, A.D., Zhao, X., Malhotra, A., Porubsky, D., Rausch, T., Gardner, E.J., Rodriguez, O.L., Guo, L., Collins, R.L., Fan, X., Wen, J., Handsaker, R.E., Fairley, S., Kronenberg, Z.N., Kong, X., Hormozdiari, F., Lee, D., Wenger, A.M., Hastie, A.R., Antaki, D., Anantharaman, T., Audano, P.A., Brand, H., Cantsilieris, S., Cao, H., Cerveira, E., Chen, C., Chen, X., Chin, C.-S., Chong, Z., Chuang, N.T., Lambert, C.C., Church, D.M., Clarke, L., Farrell, A., Flores, J., Galeev, T., Gorkin, D.U., Gujral, M., Guryev, V., Heaton, W.H., Korlach, J., Kumar, S., Kwon, J.Y., Lam, E.T., Lee, J.E., Lee, J., Lee, W.-P., Lee, S.P., Li, S., Marks, P., Viaud-Martinez, K., Meiers, S., Munson, K.M., Navarro, F.C.P., Nelson, B.J., Nodzak, C., Noor, A., Kyriazopoulou-Panagiotopoulou, S., Pang, A.W.C., Qiu, Y., Rosanio, G., Ryan, M., Stütz, A., Spierings, D.C.J., Ward, A., Welch, A.E., Xiao, M., Xu, W., Zhang, C., Zhu, Q., Zheng-Bradley, X., Lowy, E., Yakneen, S., McCarroll, S., Jun, G., Ding, L., Koh, C.L., Ren, B., Flicek, P., Chen, K., Gerstein, M.B., Kwok, P.-Y., Lansdorp, P.M., Marth, G.T., Sebat, J., Shi, X., Bashir, A., Ye, K., Devine, S.E., Talkowski, M.E., Mills, R.E., Marschall, T., Korbel, J.O., Eichler, E.E., Lee, C., 2019. Multi-platform discovery of haplotype-resolved structural variation in human genomes. Nat. Commun. 10, 1784.

De Coster, W., De Rijk, P., De Roeck, A., De Pooter, T., D'Hert, S., Strazisar, M., Sleegers, K., Van Broeckhoven, C., 2019. Structural variants identified by Oxford Nanopore PromethION sequencing of the human genome. Genome Res.

Heller, D., Vingron, M., 2019. SVIM: Structural Variant Identification using Mapped Long Reads. Bioinformatics.

Zook, J.M., Hansen, N.F., Olson, N.D., Chapman, L.M., Mullikin, J.C., Xiao, C., Sherry, S., Koren, S., Phillippy, A.M., Boutros, P.C., Sahraeian, S.M.E., Huang, V., Rouette, A., Alexander, N., Mason, C.E., Hajirasouliha, I., Ricketts, C., Lee, J., Tearle, R., Fiddes, I.T., Barrio, A.M., Wala, J., Carroll, A., Ghaffari, N., Rodriguez, O.L., Bashir, A., Jackman, S., Farrell, J.J., Wenger, A.M., Alkan, C., Soylev, A., Schatz, M.C., Garg, S., Church, G., Marschall, T., Chen, K., Fan, X., English, A.C., Rosenfeld, J.A., Zhou, W., Mills, R.E., Sage, J.M., Davis, J.R., Kaiser, M.D., Oliver, J.S., Catalano, A.P., Chaisson, M.J.P., Spies, N., Sedlazeck, F.J., Salit, M., the Genome in a Bottle Consortium, 2019. A robust benchmark for germline structural variant detection. bioRxiv.

**Response to reviewers**

**Reviewer #1:**

The authors present a new algorithm named NanoVar that was built to align and call structural variants using data generated on Oxford Nanopore devices. This is much needed, as long-read aligners and variant callers are not yet adequate, and NanoVar looks promising. The authors were thorough in their manuscript, and it is well written. There are several points, many of which are practical in nature, that I believe will strengthen the manuscript. There are a few concerns as well.

Minor:

1. The authors lump insertions and translocations into a class they call "breakends". After the initial NanoVar run, could they take all "breakends" and perform a second alignment using sequence within the SV to determine whether it is a translocation, and where it came from? Or, more simply, consider secondary alignments from BLAST? I do not think this is critical for publication, but I do think it would simplify results if possible.

Reply: We thank the reviewer for this suggestion. We have implemented this subclassification in our latest VCF file (NanoVar v1.2.x). As shown in the Rebuttal Figure 1, our new generated VCF file includes a ”SV2” field in the INFO column that indicates the prediction for each breakend (BND) entry (i.e. translocation (TLO) or sequence transposition (TPO)).

Rebuttal Figure 1: Screenshots of VCF file produced by the latest NanoVar version including information on the sub-classification of BND SVs into translocations (TLO) or transpositions (TPO). This sub-classification can be found in the “SV2” field within the “INFO” column of the VCF file as indicated in red boxes on the zoomed-in image (bottom).

2. "with an increased in NanoVar's recall" -> should be "increase"

Reply: We thank the reviewer for spotting this mistake, which is now corrected.

3. It's unclear to me whether the in silico PCR length in Figure 3b is with or without the SV. The text doesn't appear to specify: "Primer sequences were designed flanking the breakend location(s) of each SV according to the reference genome, and their referenced amplicon lengths (in silico PCR length)…" I assumed that it must be the predicted length without the SV until I looked at SV 1-2. The product size is much closer to the predicted size minus the SV size. Can you clarify this in the text and figure legend?

Reply: We apologise for being unclear on this matter. The reviewer is right, the in-silico PCR length in Figure 3b refers to the expected PCR amplicon length without the SV. This length is calculated based on the GRCh38 reference genome. We have further clarified this in the manuscript on page 13: “Primer sequences were designed flanking the breakend location(s) of each SV according to the reference genome, and their referenced amplicon lengths (in silico PCR length without the SV) are recorded in Figure 3b, along with their breakend read ratios and estimated SV sizes.”, and Figure 3 legend on page 48: “(b) Table displaying the details of SVs selected for validation for Patient 1 and Patient 2. The in-silico PCR size refers to the expected size of the PCR amplicon without the SV.”

4. I believe the following section header is inaccurate because, unless I misunderstood, NanoVar actually discovered the SVs and they were validated by PCR: "NanoVar recalls SVs discovered by PCR."

Reply: We are sorry for this ambiguous header, which does not reflect the content of the section. As detailed in our manuscript on page 13: “We went on to uncover shared SVs between Patient 1 and Patient 2 amongst the selected SVs by testing the SVs reciprocally in each patient by PCR using the same primers and cycle conditions”. Therefore, we have amended the heading to: “NanoVar recalls shared SVs validated by PCR”.

5. Should be "possess" (or remove "do"): "We found out that normal HSC samples do possessed…"

Reply: We thank the reviewer for identifying this mistake, which is now corrected as advised.

Major:

1. The authors make an important point in their paper that I feel contradicts one of their previous conclusions. The authors first state that coverage >4x does not meaningfully impact NanoVars results: "These results imply that 4X is the minimal coverage required for optimal NanoVar SV characterization and that increasing the coverage above 4X will not affect its performance."Based on Supplementary Figure 3b, I would generally agree, but the authors then point out that 3x-5x coverage may be insufficient to identify heterozygous SVs: "We investigated the four undetected shared-SVs and discovered that three of them (SVs 2-2, 2-3, 1-7) were in fact captured by NanoVar but did not pass the confidence score threshold of 2.6, possibly due to insufficient read depth coverage (3X to 5X) for heterozygous SV detection. The other undetected heterozygous shared- SV (SV 2-8) was not detected due to the absence of SV associated reads at its locus. This SV locus was found to be under-sequenced, covered only by two reads that likely originated from the wild-type allele in Patient 1. These results suggest that a higher depth of coverage (>4X) would be required by NanoVar to confidently and broadly capture heterozygous SVs." In my opinion, the authors need to be careful about advertising 4x as sufficient in the abstract and other text. They should clarify that 4x is sufficient to identify homozygous mutations, and whether that applies at the "clinical" threshold of 2.6, or the "standard" 1.4. These details are important for end users. This should also be clarified in the discussion.

Reply: We thank the reviewer for recognising this contradiction and we understand that it will confuse or mislead end users. According to the simulation benchmark data we showed, we can only claim that 4X depth is enough for detecting homozygous SVs. This should have been applicable to both thresholds of 2.6 and 1.4 as these values were derived from 4X depth simulation datasets. In the revised manuscript, we have resolved the contradiction by clarifying that 4X depth is only enough for detecting homozygous SVs, whereas 8X depth would suffice for detecting heterozygous SVs as suggested from additional heterozygous simulation experiment (new Fig. 2b). This clarification can be found on page 5: “It is optimized to work with shallow long-read WGS data at a minimum sequencing depth of 4X (12 gigabases (Gb) for homozygous SVs and 8X (24 Gb) for heterozygous SVs…”. Also, in the latest improved version of NanoVar (v1.2.7), the threshold scores have been adjusted to 1.0 to accommodate the new neural-network model.

2. I have a concern about masking repetitive sequences in the BLAST database. My understanding is that repetitive sequences were historically masked because short-read technologies are inherently incapable of handling them. Part of the promise of long-read technologies is the ability to resolve many of these regions. I think this is biologically important because there are many repeat expansion diseases (e.g., Huntington's, ALS/FTD, spinocerebellar ataxias, etc.). There is even a repeat expansion implicated in Alzheimer's disease. Would repeat masking prevent NanoVar from identifying those repeats? I believe it would. e.g., if you look at the 'GGGGCC' repeat from C9orf72 in hg38, you will see that it is in lower-case, which I believe BLAST will treat as masked.

Reply: This is an important point that we had overlooked, and we thank the reviewer for raising this concern and explaining the importance of studying repetitive sequences in diseases. In the NanoVar pipeline, a WindowMasker counts file is required by HSBLASTN aligner during the read mapping process. However, this option affects only simple repeats. Indeed, despite WindowMasker, we do not observe any effect on read mapping or SV detection in our new simulated datasets, where the majority of SVs are positioned in various types of repetitive regions. Here, we observed that NanoVar was able to recall most of these SVs with a higher sensitivity than current tools (Fig. 2e and 2f, Supplementary Fig. 3). Hence, NanoVar is still applicable to characterize SVs within repetitive regions despite the usage of WindowMasker.

3. It's not clear to me how "genomic location" was determined when benchmarking NanoVar to other algorithms. I believe the section titled "Performance evaluation in simulation datasets" is where this is briefly described. How much error was allowed for mutations to be called the same? i.e., how much error in the breakpoint location and size? This is an important consideration in SV calling. Perhaps the other callers identified the "same" mutations, but with less precision on the location?

Reply: We regret for being unclear about the evaluation criteria. We allow a 400 bp error distance from the coordinates of a ground-truth SV breakend, creating an 800 bp region. We treat each SV breakend region as an individual true event and therefore do not consider the size of an SV. For each tool we are testing, we extracted the breakend coordinates of all the SVs predicted in their VCF file. In order to justify a breakend match, the predicted breakend coordinate must fall within a ground-truth SV breakend region. We have added these clarifications in the revised manuscript on page 8: “To evaluate predicted SVs fairly, we segregated each ground-truth SV into its breakend coordinates and allow a 400 bp error distance around it to create an 800 bp window. Each DEL, INV and DUP SV possesses two breakends, while each INS and BND SV possesses one breakend. SVs predicted by each tool were also segregated to SV breakends and intersected with the ground-truth breakend regions. A hit will be considered if a predicted SV breakend falls within any ground-truth breakend region.”. An illustration of our benchmarking strategy is available online at: https://github.com/benoukraflab/nanovar/blob/master/scripts/benchmark_evaluation_exa mple.txt.

4. I see the authors provide source code and great documentation for NanoVar on github. It is important to provide all scripts and data used to generate all data. I see the simulated datasets, but it is not obvious to me where I could find scripts to rerun all analyses.

Reply: We thank the reviewer for this suggestion. We have provided the scripts used for simulated data generation and scripts for all analysis on GitHub

(<https://github.com/benoukraflab/nanovar/tree/master/scripts>).

5. I do not see an explanation for how the two confidence thresholds, 1.4 and 2.6, were determined, which is important for readers to know. More importantly, however, is whether those confidence scores are ubiquitous across situations, or are they a result of training on the neural network? i.e., can I simply download NanoVar and rely on those thresholds, or will I need to define new thresholds?

Reply: Both thresholds were derived through the testing of NanoVar on simulated data. The threshold 1.4 was observed as the optimal score for the balance of both precision and recall which constitutes the highest F1 score, whereas 2.6 was the score where precision was found to plateau close to the maximum (>0.97). The threshold 2.6 was noted to allow more stringency in precision with the cost of recall when running on real data. These thresholds function to enrich true SVs from false SVs caused by sequencing errors. Assuming that the characteristics of false SVs in the simulations are not vastly different from that of real data (as we used real sequencing reads to model the errors in read simulation), we believe that the thresholds will be applicable in real data. Moreover, we tested the sensitivity of our new model (v1.2.7, with threshold of 1.0) on a real benchmark dataset (Parikh et al., 2016) and showed good recall comparable to SVIM (Supplementary Fig. 4).

Although NanoVar is provided with a universal model and threshold score, instructions and scripts required for building a custom neural-network model is available here: (https://github.com/benoukraflab/nanovar/blob/master/scripts/NNtrain_tutorial.md). We have addressed this issue in the discussion section of the manuscript (Page 16 and 17): “Although we propose the confidence score of 1.0 as a default threshold for running NanoVar, there might be situations where it is less effective and requires the re-training of the neural network model. The threshold score was observed as the optimal score for the balance of both precision and recall in simulated experiments. Thus, the effectiveness of the threshold is dependent on the resemblance of data characteristics between simulated and real environments, which may be quite different in certain situations. Such cases may include the usage of long-reads from new sequencing technologies utterly different from ONT or PacBio, or analyzing genomes with vastly more proportion of repetitive sequences than the human genome. In these situations, the alignment profile of each read (e.g. mismatch errors, number of multi-maps) might be exclusive and thus requires re-training for a better fit using the tutorial provided on GitHub (see Availability of data and material).”.

6. Do users need to re-train the neural network in certain situations? It would be very helpful for end users if this were briefly explained in the discussion, and particularly on github. In either case, authors should provide a brief example on github for how to train, and assess whether the training is successful.

Reply: We thanks the reviewer for this suggestion. The neural network model might need to be re-trained in certain situations such as using reads from a sequencing technology apart from ONT or Pacbio, or if the user is working on other genomes with vastly more proportion of repetitive sequences than the human genome. In these situations, the read alignment profile of each read (e.g. indel errors, e-value) might be different and thus requires re-training for a better fit. If there is a demand for such situations, we will re-train the model accordingly and add them into the distribution. Nevertheless, users can retrain and assess their model using scripts and the tutorial provided on GitHub (https://github.com/benoukraflab/nanovar/blob/master/scripts/NNtrain_tutorial.md. We thank the reviewer for this important point and we have added an explanation in the discussion (c.f. point 5).

**Reviewer #2:**

The authors discuss NanoVar, a structural variant caller specifically tailored to low-coverage long-read sequencing, which is very relevant considering high sequencing costs on these platforms. I am confident that the field of long read SV discovery hasn't saturated yet.

1. I noticed that the authors mention the definition of SVs to be larger than 50 bp in their background section but include smaller variants in their simulations, and to remove those in later evaluations since this size range is highly inaccurate. It might be a better idea to be consistent here.

Reply: We thank the reviewer for pointing this out. In our new simulated data, all SVs are larger than 50 bp in length. They were estimated by RSVSim using the SV length distribution taken from the Database of Genomic Variants (DGV).

2. The background section mentions that structural variants account for most of the genetic variation in the human population, which requires to be nuanced, as it accounts for the most genetic variation in terms of nucleotides, but not in the number of variants, which would be SNVs.

Reply: We agree on this comment and have made the amendment in the manuscript on page 4: “Structural variations are implicated in the development of many human diseases [1,2] and account for most of the genetic variation by means of nucleotides in the human population [3,4].”.

3. The authors compare the performance of their tool to other tools for SV discovery from long read sequencing using simulated data. While simulating data has its merits and provides an attractive simplification of the real world, I get the impression that the simulation used to compare the SV calling algorithms is not representative of what can be expected from actual structural variants in the human genome. The variant length and number of variants distribution used for the simulations (supplementary figure 2a) is not representative of what is seen in real human SV data, which relatively show many more smaller variants (50-100 bp) and fewer large variants (> 10 kb) (Audano et al., 2019; Chaisson et al., 2019; Zook et al., 2019). It is also not clear to me if these SVs are simulated in both heterozygous and homozygous states. I also believe the random locations of SVs are inappropriate, as real SVs are significantly more frequently found in repetitive regions. This probably explains why the short-read sequencing algorithms perform relatively well in your comparison but are never this precise in any real data.

Reply: We thank the reviewer for the concerns over our simulated data and we agree that it was not representative of actual data. In our new simulated data, we have increased the number of variants from 1,150 to 32,000 SVs per genome (20,000 DEL, 1,000 INV, 1,000 DUP and 10,000 TPO (Transpositions)) and 10,000 SVs per genome (Viral INS only). The variant length distribution is now estimated from the SV length distribution taken from the Database of Genomic Variants (DGV) which has more small variants and fewer large variants (Supplementary Fig. 2a). The variant locations are also more inclined (>0.75%) to be found in repetitive regions of the genome such as SINE and LINE elements (Fig. 2e and 2f, Supplementary Fig. 3). This was done using the “repeatBias” function in RSVSim. All our previous simulations were homozygous and we have added a separate heterozygous variant dataset in the new simulation with varying depth of coverage (Fig. 2b). We described our new simulated datasets on page 24 and 25: “The R Bioconductor package, RSVSim [41], was used to introduce novel adjacencies systematically in a reference genome to create different classes of SV. One SV simulation dataset consist of two genomes where one has 32,000 SVs of deletions (20,000), balanced inversions (1,000), single tandem duplications (1,000) and genomic sequence transpositions (10,000), and the other has 10,000 SVs containing only viral insertions which mimics novel insertions. The amounts for each SV were based approximately on SV occurrence reported by Chaisson et al. [43]. SV lengths were estimated by the estimateSVSizes() function in RSVSim, which takes reference from the Database of Genomic Variants (DGV). SVs were inclined to be positioned in repetitive regions using the “repeatBias” parameter in RSVSim.”.

4. Given that outdated base calls were used to provide read features for the simulation of reads these are unlikely to represent reads which are obtained using current basecalling versions.

Reply: We thank the reviewer for raising this concern. Although we agree that the simulations may not represent reads base-called by the current base-callers such as Guppy, we believe that the simulations are still applicable for benchmarking. Most of the tools were distributed before Guppy’s released and thus should be able to cope with Albacore’s base-calling. Also, the base-calling improvement of current base-callers does not affect read mapping greatly, at least for our own data (shown below). We base-called our patient data using Guppy (v3.2.4) and compared its mapping statistics with previous Albacore base-calling (mapped using minimap2). Here is the comparison table of mapping statistics between Albacore and Guppy basecaller on two patient WGS data:

| Sample | No. of reads basecalled | | No. of reads mapped | | Percentage of  bases mapped | | Mismatch error rate | |
| --- | --- | --- | --- | --- | --- | --- | --- | --- |
|  | Albacore | Guppy | Albacore | Guppy | Albacore | Guppy | Albacore | Guppy |
| Patient 1 | 1793667 | 1795879 | 1599641  (89.2%) | 1602857  (89.3%) | 90.9% | 91.5% | 1.60E-  01 | 1.55E-  01 |
| Patient 2 | 1939432 | 1939856 | 1745806  (90.0%) | 1750558  (90.2%) | 91.2% | 91.9% | 1.83E-  01 | 1.75E-  01 |

Rebuttal Table 1: Mapping statistics of reads basecalled by Albacore or Guppy for samples Patient 1 and Patient 2. For Guppy basecalling in Patient 1, only reads generated by ONT 1D protocol were basecalled by Guppy, while the 2D reads remained basecalled by Albacore or Metrichor.

5. Due to these limitations, I recommend the authors to also do their comparison using real variant data, for example from the GIAB sequencing and SV identification study (Zook et al., 2019), by downsampling a deeply long-read sequenced genome or by simulating reads from a human genome assembly.

Reply: We thank the reviewer for the recommendation to compare using real data. We wanted to use the benchmark data from Zook et al., 2019; however, upon manual inspection of their truth set SVs on the genome browser after read mapping, we could not find concordance for several predicted SVs (Rebuttal Fig. 2). We are currently trying to get in touch with the authors to better understand their dataset. Alternatively, we performed the comparison using the benchmark sample from Parikh et al., 2016 (2676 DEL and 68 INS in NA12878) (Supplementary Fig. 4).

 Rebuttal Figure 2: Screenshots of Integrative Genomics Viewer (IGV) of read coverages and alignments of

three benchmark homozygous deletions in HG002 sample. In these three examples (A, B and C), a large

amount of reads cover the region supposed to be deleted in a homozygous state (marked in red). The

deletion size and genomic coordinates are labelled on top of each sub-figure. These three deletions were

taken from “HG002_SVs_Tier1_v0.6.vcf” and have the FILTER “PASS” and genotype of “1/1”, homozygous

(ftp://ftp-trace.ncbi.nlm.nih.gov/giab/ftp/data/AshkenazimTrio/analysis/NIST_SVs_Integration_v0.6/). All 29

million PacBio reads (ftp://ftptrace.

ncbi.nlm.nih.gov/giab/ftp/data/AshkenazimTrio/HG002_NA24385_son/PacBio_MtSinai_NIST/PPacBi_f

asta/) were mapped to GRCh37 reference genome by Minimap2. The BAM file was converted to BED file for

read alignment visualization on IGV by BEDTools. The BED file was converted to BEDGraph format by

BEDTools and subsequently to bigWig format by bedGraphToBigWig for coverage visualization on IGV.

I would also encourage the authors to use a specifically-tailored algorithm for comparing their truth and test sets in precision-recall assessment, such as truvari (https://github.com/spiralgenetics/truvari). It is not entirely clear how you used bedtools and scipy for these calculations.

Reply: We thank the reviewer for this advice. We tried using truvari but unfortunately, this software is limited to compare only SVs with sizes such as deletion and insertion, leaving out breakends such as translocation and single breakend inversion. Moreover, it has little flexibility in parsing the format of a VCF file which is inconsistent across the tools, resulting in different data being extracted and unfair comparison. To reply to the reviewer’s concern about the lack of clarity regarding how we performed our comparison, we further detailed our workflow in our manuscript and on our GitHub page. Our strategy is to define each ground-truth SV breakend as an individual true event. A 400 bp error distance from the coordinate of an SV breakend was used to create an 800 bp SV breakend window. For each tool we are testing, we extracted the breakend coordinates of all the SVs predicted in their VCF file. In order to justify a breakend match, the predicted breakend coordinate must fall within a ground-truth SV breakend region. We have added these clarifications in the revised manuscript on page

8: “To evaluate predicted SVs fairly, we segregated each ground-truth SV into its breakend coordinates and allow a 400 bp error distance around it to create an 800 bp window. Each DEL, INV and DUP SV possesses two breakends, while each INS and BND SV possesses one breakend. SVs predicted by each tool were also segregated to SV breakends and intersected with the ground-truth breakend regions. A hit will be considered if a predicted SV breakend falls within any ground-truth breakend region.”. An illustration on how the evaluation was done can be accessed from our GitHub page: https://github.com/benoukraflab/nanovar/blob/master/scripts/benchmark_evaluation_exa mple.txt. It seems to me that the number of SVs identified in the patients (7000 and 7761) is much lower than the number of SVs which is expected from human genome sequencing. Could the authors comment on this discrepancy?

Reply: The lower number of SVs was due to our stringent threshold score (2.6), initially chosen to achieve higher precision in clinical data. We are able to identify more SVs if we lower this threshold. In our new model, with threshold score at 1.0 and minimum read depth of 2, we were able to identify more confident SVs in our two patient samples (14,215 SVs and 16,562 SVs).

Could the masking of repetitive regions in the HS-BLASTN step or the low coverage sequencing explain this?

Reply: We do not think the masking of repetitive regions could restrict the sensitivity as we do not observe this in the simulation data where most SVs (>75%) were located in repetitive regions (Fig. 2e and 2f, Supplementary Fig. 3; and cf. Reviewer 2 reply on point #3). A low sequencing coverage coupled with sample heterogeneity might hinder the detection of some heterozygous SVs but would still be able to capture majority of SVs as suggested in simulated data (Fig. 2b).

I wonder if 4x coverage is overly restrictive, and the performance of these tools would be much better at 8-10x coverage, while still, affordable considering increasing throughput on ONT MinION and especially PromethION. It surprises me that increasing the throughput above 4x has a neglectible impact on the performance of NanoVar (Supplementary Figure 3), and I wonder if this would be replicated with real data. I see the authors remark that a higher coverage could be necessary for heterozygous SVs (page 12, line 43), which are probably the most frequent anyway.

Reply: We agree with the reviewer that 8-10x coverage is still affordable and feasible for ONT sequencing and would highly suggest it over 4x depth sequencing as it would help with heterozygous SV detection. In the revised manuscript, we have increased the recommended sequencing depth to 8X instead.

I failed to install your software on both a Ubuntu 19.04 and a CentOS7.7. On Ubuntu the subprocess32 installation failed (_posixsubprocess.c:16:10: fatal error: Python.h: No such file or directory #include "Python.h" compilation terminated. error: command 'x86_64-linux-gnu-gcc' failed with exit status 1). I see you use Python2.7 during the installation, although it is very much recommended to move everything to Python3 as Python2 is getting deprecated by the end of this year. My CentOS7 turned out to be incompatible due to an ldd version < 2.16. It may not always be trivial or possible for users to update such components. I recommend the authors to make their tool available through bioconda, as this offers a very convenient way of installing the software in a reproducible environment on various operating systems, also for users less comfortable using the command-line.

Reply: We regret that the reviewer failed to install our software and understand the inconvenience of troubleshooting and fixing the components mentioned. As suggested, we have packaged and distributed NanoVar in Bioconda (https://anaconda.org/bioconda/nanovar) and PyPi (https://pypi.org/project/nanovar/). We hope it would resolve the reviewer’s issue and allows an easy installation for other users.

Furthermore, I have some minor comments:

-The patient genomes were base called using albacore, suggesting that more recent basecalling using guppy could further improve results, as substantial improvements have been made to its accuracy. I wholeheartedly understand that it's impossible to keep up with the speed of software developments, but it could be interesting to see how the results are with a modern basecaller.

Reply: We thank the reviewer’s advice and understanding. We agree that using Guppy would improve read quality and mapping, but as mentioned by the reviewer, it is difficult to keep up with it in this project. Moreover, we have 2D long-read ONT data in one of our patient samples which cannot be base-called using Guppy.

-It might be interesting to include the SV caller SVIM (Heller and Vingron, 2019) in the comparison, which we have seen is also rather sensitive, and probably has a higher rate.

Reply: We thank the reviewer for informing us about SVIM. We have included SVIM in our comparison.

-I wonder if using Sniffles with -s 2 is overly restrictive for this dataset, as it can be expected that some structural variants will only be supported by a single read in such low coverage data.

Reply: We were also afraid of it restricting Sniffles’ performance and agree that some breakends will be supported by only one read. However, Sniffles yielded a high amount of false positives when we reduce it to –s 1 (Rebuttal Fig. 3), and hence, to allow a fair comparison, we kept it at –s 2 for better precision.

Rebuttal Figure 3: Precision and recall of SVs called by Sniffles using read support setting of -s 1 (red) and - s 2 (blue). The dataset used was a simulated homozygous SV genome with 42,000 SVs (the same dataset as Sim1 in Figure 2a of manuscript).

-The supplementary table 8 mentions alignment with bwa mem, which is appropriate but not optimal and the authors might want to consider replacing the aligner with minimap2, but as this is not the aligner used for the variant calling this wouldn't affect any performance results.

Reply: We thank the reviewer for his suggestion. We have rerun the alignments using minimap2 in this new version of our manuscript.

-While the authors did not mention this, I assume your tool is equally well applicable to PacBio SMRT sequencing?

Reply: NanoVar is trained and optimized for ONT sequencing. Its neural-network model was designed to work with the profiles of ONT read alignment. Nonetheless, when tested on 4X and 8X PacBio real data, we observed relatively good SV recall compared to other tools (Supplementary Fig. 4) However, we believe there is more space for improvement and will build and include a separate model for PacBio reads in a future release.

-Finally, you might find the SV caller comparison in our paper on SVs in PromethION sequencing interesting (De Coster et al., 2019).

Reply: We thank the reviewer for directing us to this paper, which was helpful to compare long-read low vs high sequencing depth SV detection tools. We discussed differences on these two strategies on the results section, page 9: “To find out if a higher sequencing depth might improve performance, we tested the 3GS tools at sequencing depths of 8X and 12X for heterozygous SVs and observed a comparable increase in recall for all tools (Fig. 2b). Despite the increase in recall, F1 score improvement was only seen in NanoVar and NanoSV as the other tools had a decrease in precision as coverage increases. The performance of NanoSV might continue to improve with an increasing depth of coverage as it was previously observed to perform better with high coverage (79X) ONT real dataset [28]”.

I came across three typos:

-Page 6 line 31: simulated dataset -> simulated datasets

-Page 9 line 9: an increased -> an increase

-Page 13 line 6: do possessed -> do possess

Reply: We thank the reviewer and acknowledge these grammar mistakes. We have corrected them in the manuscript.

**2nd round**

**Reviewer 1**

I appreciate the authors' sincere considerations to my concerns. They have done an excellent job. I have no major concerns remaining.

Regards,

Mark Ebbert

**Reviewer 2**

The authors present their revised version of their manuscript on Nanovar. In general, I am satisfied with their additional analyses, clarifications and in-depth responses to my comments and suggestions, and I only have some minor comments. The performance of Nanovar is impressive, and installation with conda was without issues and I thank the authors for making their software easily accessible.

- I believe that heterozygous SVs are more common, and homozygous variants rather an exception, for example with very common variants or in the context of genetic research in consanguineous families. If the authors agree I would suggest to slightly rewrite some sentences to focus first on heterozygous variants, of which I think the performance is the most relevant to the readers. In that context, I would also recommend discussing 8x coverage more prominently and mention 4x coverage as "sufficient for homozygous variants only", or remove it altogether in the interest of clarity.

- In addition to making the simulations more realistic, the authors have now included an evaluation with a real PacBio dataset, however with an imperfect set of benchmarking variants (only 2,676 deletions and 68 insertions). I agree that finding good ground truth data is hard, and the lack of concordance with the GIAB set is worrying. I do expect readers to wonder why you chose the data from Parikh et al rather than the GIAB release, therefore a note explaining your choice might be recommended, optionally in the supplementary material. The lack of truly golden standards definitely makes the evaluation of software troublesome. Perhaps the structural variants identified by Chaisson et al., 2019 integrating multiple technologies could be suitable for further evaluation. In my opinion, further benchmarking is not critical for this publication but could nonetheless be informative. Our high coverage PromethION data from NA19240 is available and could be used to compare against the calls from Chaisson et al if the authors would like to further evaluate Nanovar on ONT data.

- Figure 1c still mentions the confidence threshold of 2.6, while Figure 3 and the text mention 1.0.

- Perhaps, given the rather poor performance in respectively precision and recall of Picky and NanoSV, it would improve the clarity if the text focusses on the comparison with Sniffles and SVIM. The other variant callers could be moved to the supplementary. These changes are however not critical at all.

**Response to reviewers**

**Reviewer 2**

The authors present their revised version of their manuscript on Nanovar. In general, I am satisfied with their additional analyses, clarifications and in-depth responses to my comments and suggestions, and I only have some minor comments. The performance of Nanovar is impressive, and installation with conda was without issues and I thank the authors for making their software easily accessible.

- I believe that heterozygous SVs are more common, and homozygous variants rather an exception, for example with very common variants or in the context of genetic research in consanguineous families. If the authors agree I would suggest to slightly rewrite some sentences to focus first on heterozygous variants, of which I think the performance is the most relevant to the readers. In that context, I would also recommend discussing 8x coverage more prominently and mention 4x coverage as "sufficient for homozygous variants only", or remove it altogether in the interest of clarity.

Reply: We agree that heterozygous SVs might be more common if we are looking at consanguineous families. However, we believe that homozygous SVs are also very common across individuals in a population where ancestries are diverse. Ultimately, it depends on the user’s research goals and we believe that both types of SVs are important as of now.

We had mentioned the coverage requirement for each SV zygosity in the introduction on page 5: “It is optimized to work with shallow long-read WGS data at a minimum sequencing depth of 4X (12 gigabases (Gb)) for homozygous SVs and 8X (24 Gb) for heterozygous SVs,…” and also in the discussion on page 16: “Results from both simulated and real data also suggested that 4X sequencing depth may be suboptimal for comprehensive heterozygous SV discovery and would recommend a higher sequencing depth of 8X.”.

- In addition to making the simulations more realistic, the authors have now included an evaluation with a real PacBio dataset, however with an imperfect set of benchmarking variants (only 2,676 deletions and 68 insertions). I agree that finding good ground truth data is hard, and the lack of concordance with the GIAB set is worrying. I do expect readers to wonder why you chose the data from Parikh et al rather than the GIAB release, therefore a note explaining your choice might be recommended, optionally in the supplementary material. The lack of truly golden standards definitely makes the evaluation of software troublesome. Perhaps the structural variants identified by Chaisson et al., 2019 integrating multiple technologies could be suitable for further evaluation. In my opinion, further benchmarking is not critical for this publication but could nonetheless be informative. Our high coverage PromethION data from NA19240 is available and could be used to compare against the calls from Chaisson et al if the authors would like to further evaluate Nanovar on ONT data.

Reply: We did not use the GIAB release because we do not find concordance during our manual inspection of their true SV sites on a genome browser after read mapping. However, we would prefer to clarify and troubleshoot the issue with the authors first before disclosing the reason for our choice.

- Figure 1c still mentions the confidence threshold of 2.6, while Figure 3 and the text mention 1.0.

Reply: We thank the reviewer for pointing this mistake, we have amended the threshold of Figure 1c to 1.0.

- Perhaps, given the rather poor performance in respectively precision and recall of Picky and NanoSV, it would improve the clarity if the text focusses on the comparison with Sniffles and SVIM. The other variant callers could be moved to the supplementary. These changes are however not critical at all.

Reply: Because Picky and NanoSV are relatively popular in the community, we followed the Editor recommendation and kept the performance benchmark results of these two programs within the main figures.
